# Skeletal Stilbenolignan Enantiomers and Flavonoid Derivatives Against Depression in Mice from *Dracaena cochinchinensis* Exudates

**DOI:** 10.3390/ijms27093966

**Published:** 2026-04-29

**Authors:** Tian-Chang He, Fan Fang, Wei-Fen Li, Wen-Jing Yao, Jing-Jing Qi, Yong-Xian Cheng

**Affiliations:** 1 Guangdong Provincial Key Laboratory of Chinese Medicine Ingredients and Gut Microbiomics, Institute for Inheritance-Based Innovation of Chinese Medicine, School of Pharmacy, Shenzhen University Medical School, Shenzhen University, Shenzhen 518060, China; htianchang@163.com (T.-C.H.); ffan62@126.com (F.F.); liweifen@szu.edu.cn (W.-F.L.); yaowenjing@stu.cdutcm.edu.cn (W.-J.Y.); 2 Jiangxi Province Key Laboratory of Natural and Biomimetic Drugs Research, College of Pharmacy, Jiangxi Normal University, Nanchang 330022, China

**Keywords:** *Dracaena cochinchinensis* exudates, stilbenolignan, flavonoid, depression

## Abstract

A pair of enantiomeric stilbene–lignan hybrids, (−)- and (+)-dracaenolignan A (**1**), and four new flavonoid derivatives, xuejieins F–I (**2**–**5**), were isolated from *Dracaena cochinchinensis* exudates. Compound **1** features an unprecedented carbon skeleton with a 6/6/5/6-fused ring system and four consecutive chiral carbons. Their structures were clarified by spectroscopic data, theoretical NMR calculations, and ECD calculations. Furthermore, a potential biogenetic pathway for **1** is put forward. Biological evaluations inspired by the chemical defense of *D. cochinchinensis* revealed that (−)-**1** significantly alleviates LPS-induced neuroinflammation and depression-like behaviors, suggesting potential antidepressant lead structure.

## 1. Introduction

Stilbenes, a significant class of naturally occurring phenolic compounds that are extensively prevalent across diverse plant species such as Vitaceae, Fabaceae, and Dipterocarpaceae, are collectively defined as monomers and polymers featuring a characteristic C6–C2–C6 skeleton [1]. Stilbenolignans, a distinct class of plant secondary metabolites, arise from the oxidative coupling of a stilbene unit (C6–C2–C6) with a phenylpropanoid unit (C6–C3), and constitute a group of non-conventional lignans [2]. To date, over 1041 stilbene compounds have been reported [3]. Recent pharmacological investigations reveal that they present a wide array of bioactivities encompassing antioxidant, anti-bacterial, anti-inflammatory, neuroprotective, and antitumor activities [4,5,6,7,8,9]. Consequently, stilbenes have attracted extensive research interest from pharmacologists and natural product chemists.

**Figure 1:**
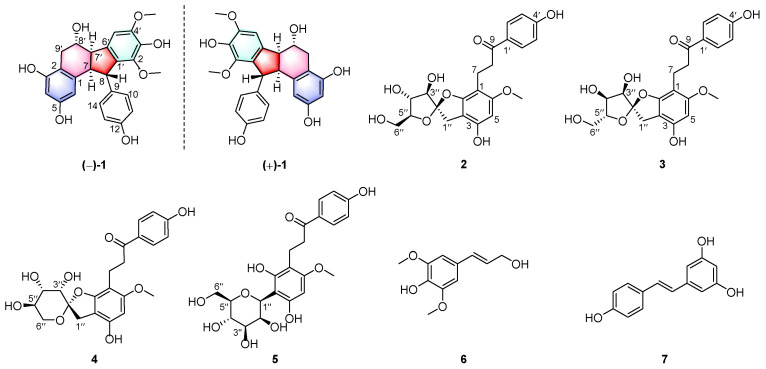
The chemical structures of compounds **1**–**7**.

The resins obtained from *Dracaena cochinchinensis* S. C. Chen, commonly referred to as Chinese dragon’s blood, are mainly distributed in southwest China, particularly in Yunnan and Guangxi provinces [10]. Dragon’s blood, a traditional Chinese medicine, exhibits multiple therapeutic effects, such as promoting circulation, eliminating blood stasis, arresting bleeding, alleviating pain, healing sores, and facilitating muscle regeneration. Phytochemical studies have identified a diverse array of constituents, including phenols, terpenoids, steroids, saponins, stilbenoids, alkaloids, and polysaccharides [11,12,13]. Modern pharmacological research shows it has extensive effects including wound-healing promotion, anti-inflammatory, antitumor, anti-bacterial, neuroprotection, and antithrombotic effects, which represent promising molecular scaffolds for intervening in neuroinflammation-associated depression [11,12,13,14]. Depression is often accompanied by chronic neuroinflammation and the dysregulation of cerebral blood flow, providing a theoretical basis for investigating the resin’s components in this context [15,16,17]. Previous studies have indicated that phenolic constituents, particularly flavonoids and stilbenoids, are the major bioactive components of this resin [18,19]. To discover structurally intriguing and biologically significant natural compounds from the red resins of *D. cochinchinensis* [20,21], a pair of novel enantiomeric stilbenolignans, (−)- and (+)-dracaenolignans A (**1**), and four new flavonoid derivatives, xuejieins F–I (**2**–**5**), together with two known compounds, sinapyl alcohol (**6**) and resveratrol (**7**) (Figure 1), were isolated. In particular, the discovery of structurally novel and biologically intriguing stilbenes, (−)- and (+)-**1,** will offer new perspectives on the resins of *D. cochinchinensis.* Herein, the isolation, structural characterization, biosynthetic postulation, and antidepressant activities are detailed.

## 2. Results and Discussion

### 2.1. Structure Elucidation of the Compounds

Dracaenolignan A (**1**), a reddish-brown gum, was established as C_25_H_24_O_7_ with 14 degrees of unsaturation (DOUS) according to the (−)-HRESIMS (*m*/*z* 435.1447 [M − H]^−^, calcd for C_25_H_23_O_7_ 435.1449) and ^13^C NMR data. The ^1^H NMR spectrum (Table 1) of **1** shows two methoxy groups (*δ*_H_ 3.23, s, 2′-OCH_3_; *δ*_H_ 3.89, s, 4′-OCH_3_), a 1,4-disubstituted benzene ring (*δ*_H_ 7.01, d, *J* = 8.5 Hz, H-10, 14; *δ*_H_ 6.76, d, *J* = 8.5 Hz, H-11, 13), a 1,2,3,5-tetrasubstituted benzene ring (*δ*_H_ 6.13, d, *J* = 2.3 Hz, H-4; *δ*_H_ 5.88, d, *J* = 2.3 Hz, H-6), and a 1,2,3,4,5-pentasubstituted benzene ring (*δ*_H_ 6.84, s, H-5′). The ^13^C NMR and DEPT-135 spectra (Table 1) show 25 carbon resonances, corresponding to two methoxy groups, one methylene, 11 methines (7 olefinic and 4 aliphatic including one oxygenated), and 11 non-protonated carbons (11 olefinic including six oxygenated). The aforementioned functionalities accounted for twelve out of the fourteen DOUs, and the remaining two additional rings thus implied that **1** possesses a pentacyclic ring system.

**Figure 2:**
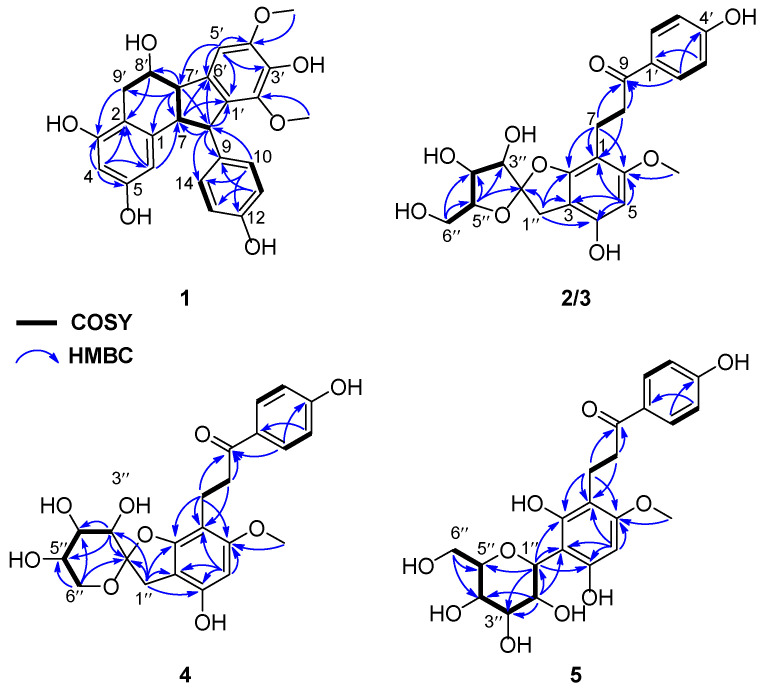
^1^H–^1^H COSY and key HMBC correlations of compounds **1**–**5**.

The planar structure of **1** was mainly determined by 2D NMR experiments. The ^1^H–^1^H COSY correlations (Figure 2) of H-7/H-8 and H-10 (14)/H-11 (13), in combination with the HMBC correlations (Figure 2) of H-4/C-2 (*δ*_C_ 113.7) and C-6 (*δ*_C_ 107.5), H-6/C-2 and C-7 (*δ*_C_ 54.9), H-10/C-8 (*δ*_C_ 58.4), C-12 (*δ*_C_ 156.8), and C-14 (*δ*_C_ 130.4), H-11/C-9 (*δ*_C_ 138.0) and C-13 (*δ*_C_ 116.1), allowing the establishment of a stilbene moiety. The ^1^H–^1^H COSY correlations of H-7′/H-8′/H_2_-9 and the HMBC correlations of 2′-OCH_3_/C-2′ (*δ*_C_ 145.5), 4′-OCH_3_/C-4′ (*δ*_C_ 149.7), H-5′/C-1′ (*δ*_C_ 136.7), C-3′ (*δ*_C_ 139.8), and C-7′ (*δ*_C_ 51.9) revealed a phenylpropanoid moiety. Furthermore, the ^1^H–^1^H COSY correlations of H-7/H-7′, the HMBC correlations of H-7/C-1′ and C-6′, H-8/C-1′, C-2′, and C-6′, and the two remaining DOUs demonstrated that the stilbene and phenylpropanoid moiety are connected by fusing an indene ring composed of C-1, C-2, C-9′, C-8′, C-7′, C-6′, C-1′, C-8, and C-7. Thus, the planar structure of **1** was assigned as a novel stilbenolignan, characterized by a 6/6/5/6-fused framework.

**Figure 3:**
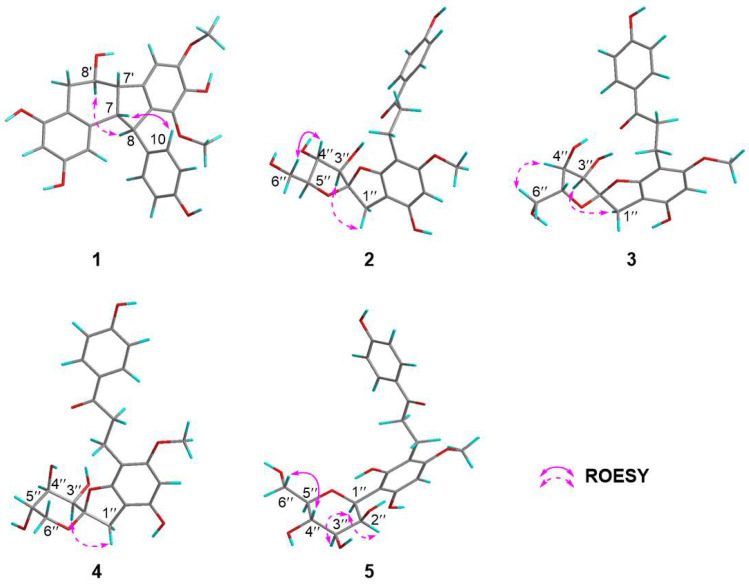
Key ROESY correlations of compounds **1**–**5**.

**Figure 4:**
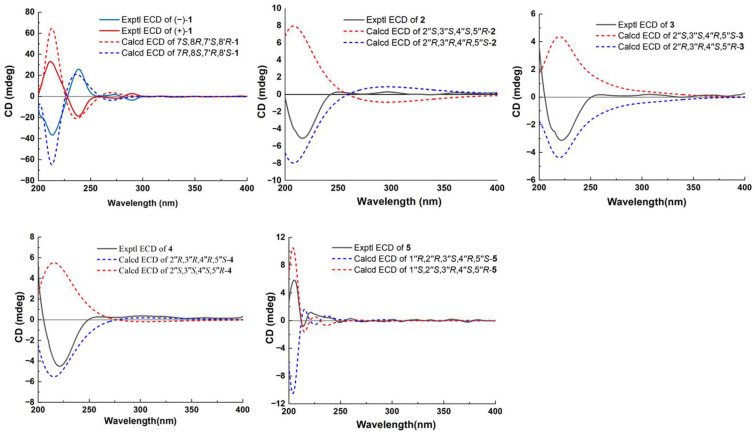
Experimental and calculated ECD spectra of compounds **1–5**.

The relative configuration of **1** was established by detailed interpretation of the ROESY spectrum (Figure 3). The ROESY correlations of H-10/H-7 and H-8/H-8′ indicated that H-7 and *p*-hydroxybenzyl are on the same side, H-8 and H-8′ are on the other side. However, the relative configuration of the C-7′ could not be determined directly from the ROESY data due to H-7 and H-7′ being in adjacent positions. Then, theoretical NMR calculations and DP4+ analysis were applied to distinguish the two possible isomers (7S*,8R*,7′S*,8′R* and 7S*,8R*,7′R*,8′R*) for **1**, and the calculation results showed that the 7S*,8R*,7′S*,8′R*-isomer is the most likely candidate with 100.00% probability (Figure S44). Hence, the relative configuration of **1** was defined as 7S*,8R*,7′S*,8′R*. Notably, the close-to-zero specific rotation value of **1** led to the suspicion that **1** might exist as a racemic mixture. We subsequently resolved it into two optically pure enantiomers, (−)-**1** and (+)-**1** in a 1:1 ratio, by chiral HPLC. Finally, the absolute configurations of (−)-**1** and (+)-**1** were assigned as (7*R*,8*S*,7′*R*,8′*S*) and (7*S*,8*R*,7′*S*,8′*R*), respectively (Figure 4), based on time-dependent density functional theory (TDDFT) ECD calculations and comparison of the experimental CD spectrum with the calculated one.

Xuejiein F (**2**), obtained as a white solid, was deduced as C_22_H_24_O_9_ with 11 DOUS according to the HRESIMS and ^13^C NMR data. The ^1^H NMR spectrum (Table 2) of **2** reveals the presence of a 1,4-disubstituted (*δ*_H_ 7.90, d, *J* = 8.8 Hz, H-2′, 6′; *δ*_H_ 6.82 d, *J* = 8.8 Hz, H-3′, 5′) and a 1,2,3,4,5-pentasubstituted benzene rings signals (*δ*_H_ 5.95, s, H-5), one oxygenated methylene signal (*δ*_H_ 3.73, dd, *J* = 11.8, 3.4 Hz, Ha-6″; *δ*_H_ 3.66, dd, *J* = 11.8, 7.2 Hz, Hb-6″), and one methoxy groups (*δ*_H_ 3.69, s, 6-OCH_3_). The ^13^C NMR spectrum (Table 2) contains 22 resonances attributable to one methoxy, four methylene including one oxygenated, eight methines (five olefinic and three aliphatic), and nine non-protonated carbons (one carbonyl, seven olefinic, and one aliphatic). The two benzene rings and one carbonyl group account for nine DOUs, thus requiring **2** to be a tetracyclic compound.

The ^1^H–^1^H COSY spectrum (Figure 2) of **2** reveals correlations of H_2_-7/H_2_-8, H-2′/H-3′, H-5′/H-6′, and H-3″/H-4″/H-5″/H_2_-6″. The linkages of four units involving the quaternary carbons and the other functionalized groups were elucidated mainly by analysis of the HMBC data (Figure 2), which reveal the correlations of H-7/C-2 (*δ*_C_ 159.4), C-6 (*δ*_C_ 159.9), C-9 (*δ*_C_ 202.2), H-8/C-1 (*δ*_C_ 104.0), H-5/C-1, C-3 (*δ*_C_ 104.1), C-4 (*δ*_C_ 153.0), C-6, and H-2′/C-9, C-4′ (*δ*_C_ 164.1) allowing to construct the skeleton of flavone. The HMBC correlations of H-6″/C-4″ (*δ*_C_ 76.2), C-5″ (*δ*_C_ 84.2), H-5″/C-2″ (*δ*_C_ 116.8), C-3″ (*δ*_C_ 81.3), C-4″, H-3″/C-1″, C-2″, and H-1″/C-2, C-2″, C-3″ indicated that C-5″ forms a five-membered ring with C-2″ through an “O” bridge, and C-2″ forms a ring with C-2 through an “O” bridge. The mutual coupling constants of *J*_3″,4″_ = 8.3 Hz and *J*_4″,5″_ = 7.2 Hz and the ROESY corrections (Figure 3) of H_2_-1″/H-3″, H-4″/H-6″ indicated that H-3″ and H-4″ are on the different side, H-3″ and H-5″ on the same face. Thus, the relative configuration of **2** was assigned. To clarify the absolute configuration, computational methods were carried out. ECD calculations revealed that the calculated spectrum of (2″*R*,3″*R*,4″*R*,5″*S*)-**2** agrees well with the experimental one, allowing the unambiguous assignment of **2**′s absolute stereochemistry (Figure 4).

Xuejiein G (**3**) was revealed to share the same molecular formula, C_22_H_24_O_9_, as **2**, based on analysis of their HRESIMS and NMR spectra. An analysis of their 1D and 2D NMR data revealed that they have the same planar structure, suggesting that they are stereoisomers with the difference residing in the configuration of the sugar moiety, as evidenced by chemical shift differences from C-1″ to C-6″. Furthermore, the ROESY correlations (Figure 3) of H-1″/H-3″, H-4″/H-6″, together with the coupling constants *J*_3″,4″_ = 3.8 Hz and *J*_4″,5″_ = 6.3 Hz, allowed the relative configuration of **3** to be determined, indicating that H-3″, H-4″ are on the same side and H-4″, H-5″ are on the opposite side. Likewise, the absolute configuration of **3** was assigned as 2″*R*,3″*R*,4″*S*,5″*R* by the well-matched TDDFT calculated and experimental ECD curves (Figure 4).

Xuejiein H (**4**) possesses the molecular formula C_22_H_24_O_9_ based on analysis of its HRESIMS and ^13^C NMR data. Comparing the NMR data (Table 3) of **4** with those of **3** demonstrated that they are structural analogs. The major difference between them was that a six-membered ring was connected to the C-2″, rather than a five-membered ring in **3**, which was verified by the HMBC correlations (Figure 2) of H-6″/C-2″, C-4″, C-5″. The ROESY correlations (Figure 3) of H-1″/H-3″ and the coupling constants of *J*_3″,4″_ = 2.8 Hz *J*_4″,5″_ = 9.3 Hz indicated that H-3″ and H-4″ are on the same side and H-4″ and H-5″ on the different side. Finally, the absolute configuration of **4** was determined as 2″*R*,3″*R*,4″′*R*,5″*S* by comparing the calculated ECD spectrum with the experimental one (Figure 4).

Xuejiein I (**5**) has a molecular formula of C_22_H_26_O_10_ (10 DOUs) based on its HRESIMS and ^13^C NMR data. Analysis of its ^1^H and ^13^C NMR spectra (Table 3) and HSQC and 135° DEPT spectra revealed 22 carbon resonances assignable to one methoxy, three methylenes, ten methines (five olefinic and five aliphatic including four oxygenated), and eight non-protonated carbons (one carbonyl and seven olefinic). These functional groups contribute to nine DOUs, with the remaining ones indicating that **5** possesses a tricyclic system. Comprehensive interpretation of its 1D and 2D NMR data further showed that **5** is also a flavonoid derivative. The ^1^H–^1^H COSY correlations (Figure 2) of H_2_-7/H_2_-8, H-2′/H-3′, H-5′/H-6′ and H-1″/H-2″/H-3″/H-4″/H-5″/H_2_-6″ and the HMBC correlations (Figure 2) of H-7/C-2 (*δ*_C_ 156.4), C-6 (*δ*_C_ 159.6), C-9 (*δ*_C_ 202.8), H-8/C-1 (*δ*_C_ 108.9), H-5/C-1, C-3 (*δ*_C_ 105.0), C-4 (*δ*_C_ 155.3), C-6 (*δ*_C_ 159.6), 6-OCH_3_/C-6, H-2′/C-9, C-4′ (*δ*_C_ 163.8), H-1″/C-2, C-3″ (*δ*_C_ 76.3), C-5″, and H-6″/C-4″ made it possible to construct the planar structure of **5**. As for its relative configuration, it was partially interpreted by a ROESY spectrum (Figure 3) of H-2″, H-3″/H-1″ and the coupling constants of *J*_1″,2″_ = 1.2 Hz, *J*_2″,3″_ = 3.2 Hz, *J*_3″,4″_ = 9.3 Hz, *J*_4″,5″_ = 9.3 Hz, indicating that H-1″, H-2″, H-3″, and H-5″ are spatially close and H-4″ is on the other side. The absolute configuration of **5** was further elucidated by TDDFT-ECD calculations. The calculated ECD spectrum of **5** matched well with the experimental one (Figure 4), confirming the absolute configuration as 1″*S*,2″*S*,3″*R*,4″*S*,5″*R*.

**Scheme 1:**
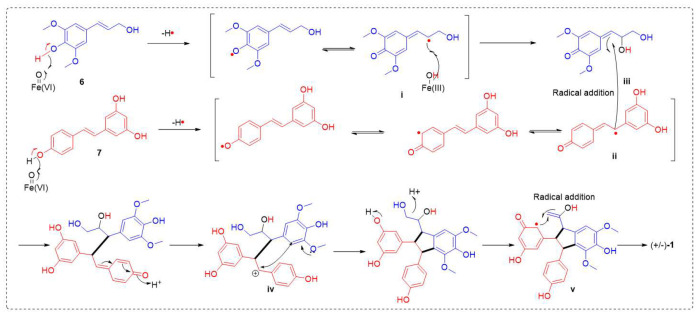
Proposed biosynthetic pathways for compound **1**.

Dracaenolignan A (**1**) is a novel skeleton stilbenolignan featuring a distinctive 6/6/5/6-fused framework with four consecutive chiral centers, making it challengeable for total chemical synthesis. Inspired by its unique scaffold, we proposed a plausible biosynthesis pathway that may originate from the co-isolated analog sinapyl alcohol (**6**) [22] and resveratrol (**7**) [23] (Scheme 1). We hypothesize that O–H activation of the phenolic hydroxyl groups in both **6** and **7**, possibly mediated by oxidative radical coupling enzymes, generating phenoxyl radicals [24]. These radicals then undergo resonance to form the allylic radicals intermediates **i** and **ii**. Among them, intermediate **i** accepts rebound from the Fe-activated hydroxyl group to yield diol intermediate **iii**, which subsequently undergoes radical addition by intermediate **ii** to form the first coupled C–C bond. The second C–C bond is presumably formed after the generation of a benzylic carbocation, which undergoes nucleophilic attack by the electron-rich aromatic ring. Following dehydration of the terminal hydroxyl group to afford a terminal double bond, the phenoxyl radical re-forms and undergo resonance to produce the allylic radical intermediate **v**. This radical attacks the terminal double bond via radical addition, thereby forming the third coupled C–C bond and ultimately affording compound **1**.

### 2.2. Biological Activity

**Figure 5:**
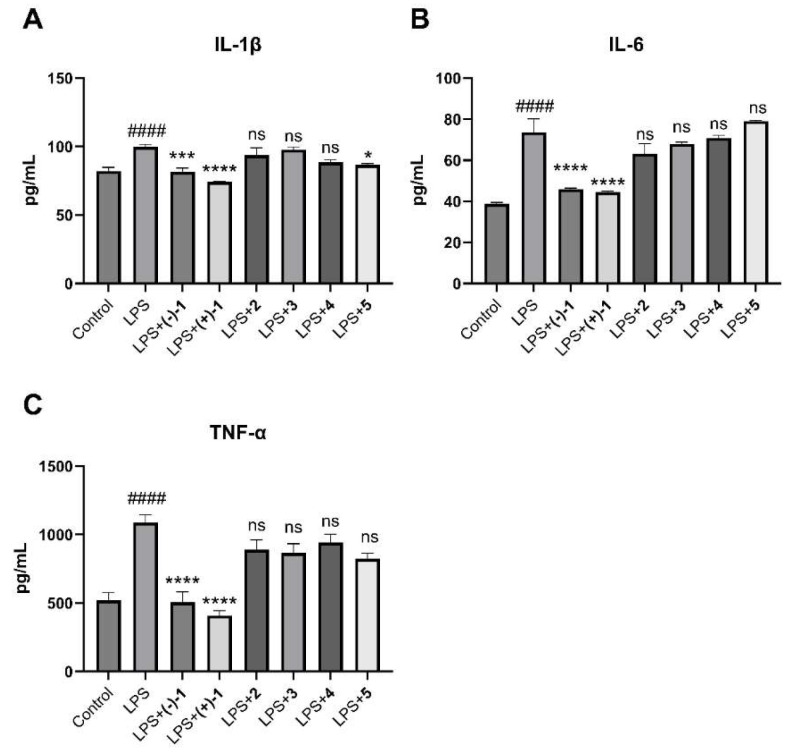
Compounds (−)-**1** and (+)-**1** attenuate LPS-induced neuroinflammation in BV2 cells in vitro. (**A**–**C**) ELISA test for (**A**) IL-1*β*, (**B**) IL-6, (**C**) TNF-*α*. Cells were treated with phosphate-buffered saline (control) or lipopolysaccharide (LPS) to induce inflammation, with or without compounds (*n* = 5 independent wells per group). Data, presented as mean ± SEM, were analyzed using GraphPad Prism 10. Statistical significance versus the control group is indicated by ^####^ *p* < 0.0001; versus the LPS group by * *p* < 0.05, *** *p* < 0.001, **** *p* < 0.0001; ns, not significant.

**Figure 6:**
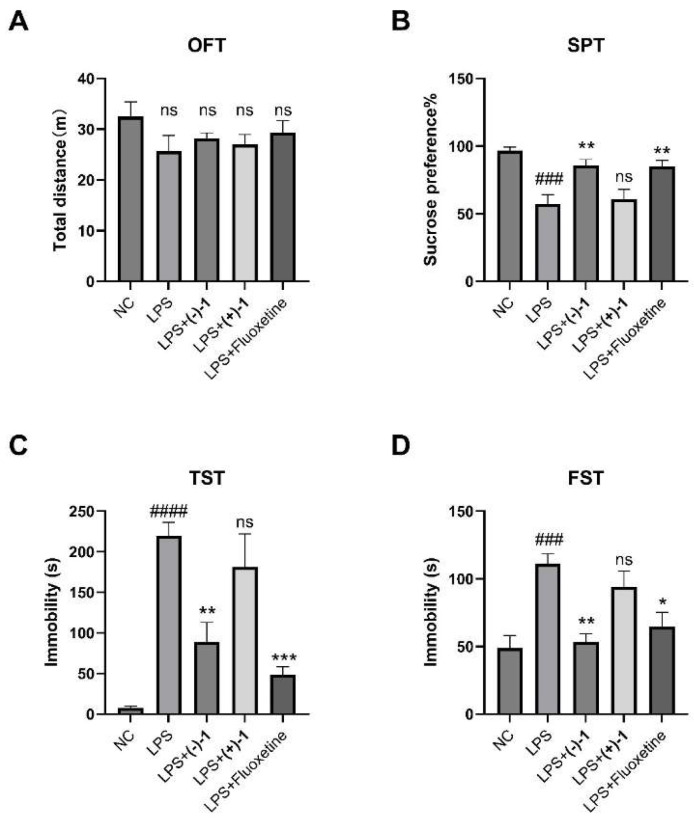
Compound (−)-**1** alleviates LPS-induced depressive-like behaviors in mice (*n* = 6 mice per group). The antidepressant effect was assessed through a battery of behavioral tests: (**A**) open field test (OFT), (**B**) sucrose preference test (SPT), (**C**) tail suspension test (TST), and (**D**) forced swimming test (FST). Mice were administered LPS to establish the depression model, with fluoxetine serving as the positive control. Data are presented as mean ± SEM and were analyzed using GraphPad Prism. Statistical significance compared to the normal control (NC) group is indicated by ^###^ *p* < 0.001 and ^####^ *p* < 0.0001; significance relative to the LPS model group is marked by * *p* < 0.05, ** *p* < 0.01, and *** *p* < 0.001; ns, not significant.

Plants produce exudates (known as resins) under different stresses to prevent themselves from injury. Therefore, it is rational to search for anti-inflammatory or antimicrobial agents from such a resource. In this context, a series of experiments were conducted to evaluate the anti-neuroinflammatory and antidepressant potential of the compounds. First, the CCK-8 assay was carried out in BV2 cells. It was found that compound **1** is toxic at higher concentrations, whereas it is not the case for compounds **2**–**5** (Figure S45). To compare the biological potency of **1**–**5** against inflammation, a further in vitro assay was performed at 1 μM, revealing that lipopolysaccharide (LPS) stimulation significantly upregulated the expression of three pro-inflammatory cytokines, namely TNF-α, IL-6 and IL-1*β*, while compound (−)-**1** and (+)-**1**, instead of compounds **2**–**5,** could attenuate these changes (Figure 5). Based on these findings, (−)-**1** and (+)-**1** were further evaluated in an LPS-induced mouse model of depression. In behavioral tests, LPS administration did not alter locomotor distance but increased immobility time and reduced sucrose preference. Treatment with (−)-**1**, not (+)-**1,** significantly increased sucrose preference and decreased immobility time (Figure 6). These results demonstrated that compound (−)-**1** effectively mitigates LPS-induced neuroinflammation and related depression-like behaviors. Of note is that these findings showed that structurally novel stilbene (**1**) might be promising in inflammation-associated depression. In addition, it remains possible that flavonoids **2**–**5** exhibit anti-inflammatory activity at higher concentration, whereas their potency might be much lower that of **1**.

## 3. Experimental Section

### 3.1. General Procedures

Optical rotation measurements were performed on an Anton Paar MCP-100 polarimeter. UV and CD spectra were obtained on a Jasco J-815 Circular dichroism spectrometer (JASCO, Japan). NMR spectroscopic data were acquired using a Bruker spectrometer (500 MHz, Karlsruhe, Germany). High-resolution electrospray ionization mass spectrometry (HRESIMS) analyses were conducted on a Shimazu LC-20 CE AB SCIEX triple TOF X500R MS spectrometer (Shimadzu Corporation, Tokyo, Japan). Column chromatography was executed utilizing MCI gel CHP 20P (75–150 μm, Mitsubishi Chemical Industries, Tokyo, Japan) and Sephadex LH-20 (Amersham Pharmacia, Uppsala, Sweden). For vacuum liquid chromatography (VLC), silica gel (300–400 mesh, Qingdao Marine Chemical Inc, Qingdao, China) was employed. Semi-preparative HPLC or preparative HPLC was performed on an Agilent 1200 liquid chromatograph (Agilent Technologies, Santa Clara, CA, USA) fitted with either an Agilent ZORBAX SB-C18 column (250 mm × 9.4 mm, i.d., 5 μm) or a YMC-Pack ODS-A column (250 mm × 10.0 mm, i.d., 5 μm). Chiral separation of enantiomers was achieved using a Daicel Chiralpak IC chiral HPLC column (250 mm × 10 mm, i.d., 5 μm). All solvents utilized for column chromatography were of either analytical grade (SCR, Sinopharm Chemical Reagent Co., Ltd., China) or HPLC grade (J&K Scientific Ltd., China).

### 3.2. Resin Material

The resin of *D. cochinchinensis* was gathered in January 2019 at a pharmaceutical factory affiliated with Guangxi University of Traditional Chinese Medicine in Nanning, Guangxi province, PR China, where it was authenticated by Mr. Xiao-Tong Huang. The voucher specimen (CHYX-0622) has been deposited at Shenzhen University, China.

### 3.3. Extraction and Isolation

The 20.0 kg dragon′s blood red powder resin was subjected to extraction with ethyl acetate at room temperature (120 L × 3, 48 h). The ethyl acetate extract (14.0 kg) was separated into six parts (Fr. A–Fr. F) on an MCI gel CHP 20P column eluting with solvent system methanol–water (15–100%).

Fr. B (720.0 g) was further fractionated on MCI gel CHP 20P using a gradient of aqueous MeOH (15–70%) to obtain five portions (Fr. B1–Fr. B5). Fr. B2 (59.0 g) was carried out on Sephadex LH-20 (MeOH), giving five portions (Fr. B2.1–Fr. B2.5). Fr. B2.2 (140 g) was separated by using an ODS medium pressure column with gradient solvent system (MeOH/H_2_O, 10–60%) to give six parts (Fr. B.2.2.1–Fr. B.2.2.6). Fr. B.2.2.1 (3.1 g) was purified by Sephadex LH-20 (MeOH), and subsequently purified by semi-preparative HPLC (MeOH/H_2_O, 12%, flow rate: 3 mL/min) to obtain compound **6** (4.4 mg, *t*_R_ = 25.3 min). Fr. B2.3 (33.3 g) was isolated by an ODS medium pressure column with gradient solvent system (MeOH/H_2_O, 10–60%) to give seven components (Fr. B.2.3.1–Fr. B.2.3.7). Fr. B.2.3.5 (5.5 g) was eluted by polyamide chromatography (MeOH/H_2_O, 10–100%) to obtain eight components (Fr. B.2.3.5.1–Fr. B.2.3.5.8). B.2.3.5.3 (1.7 g) was isolated by a solid phase extraction with CH_2_Cl_2_/MeOH (100:1 to 10:1) to afford 11 parts (B.2.3.5.3.1–B.2.3.5.3.11), and then purified by preparative and semi-preparative HPLC (MeOH/H_2_O, 45%) to obtain compounds **2** (3.1 mg, *t*_R_ = 10.3 min), **5** (16.1 mg, *t*_R_ = 12.4 min), and **4** (1.6 mg, *t*_R_ = 16.6 min). B.2.3.5.4 (1.4 g) was separated by solid phase extraction with CH_2_Cl_2_/acetone (50:1 to 1:1), and finally separated by preparative HPLC (MeOH/H_2_O, 45%, flow rate: 3 mL/min) to provide component **3** (9.9 mg, *t*_R_ = 13.0 min,). Fr. B.2.3.5.5 (615.0 mg) was purified by polyamide chromatography to obtain 14 fractions (Fr. B.2.3.5.5.1–Fr. B.2.3.5.5.14), and finally purified by semi-preparative HPLC (MeOH/H_2_O, 23%, flow rate: 3 mL/min) to obtain compound **1** (9.9 mg, *t*_R_ = 13.7 min). Fr. B2.5 (4.7 g) was subjected to a silica gel column washed with CH_2_Cl_2_/acetone (30:1 to 3:1) and subsequently purified by semi-preparative HPLC (MeOH/H_2_O, 45%, flow rate: 3 mL/min) to get compound **7** (15.8 mg, *t*_R_ = 12.4 min).

Compound (±)-**1** was resolved into a pair of enantiomers, (−)-**1** (4.0 mg) and (+)-**1** (3.9 mg), via chiral HPLC on a Daicel Chiralpak IC column eluting with *n*-hexane/ethanol (80:20).

### 3.4. Compound Characterization Data

Dracaenolignan A (**1**): Yellowish gums; UV (MeOH) *λ*_max_ (log *ε*) 282 (3.23), 200 (4.36) nm; (−)-**1**: $[\alpha {]}_{D}^{20}$ −13 (*c* 0.03, MeOH); ECD (MeOH) *λ* (Δ*ε*) 211 (−5.17), 238 (+9.83), 272 (+0.66), 288 (−1.32) nm. (+)-**1** $[\alpha {]}_{D}^{20}$ +10 (*c* 0.02, MeOH); CD (MeOH) *λ* (Δε) 211 (+18.10), 238 (−10.13), 272 (−0.76), 288 (+1.55) nm. ^1^H and ^13^C NMR data (CD_3_OD) see Table 1. HRESIMS *m*/*z* 435.1447 [M − H]^−^ (calcd for C_25_H_23_O_7_, 435.1449).

Xuejiein F (**2**): White solids; [*α*]$[\alpha {]}_{D}^{20}$ −26 (*c* 0.04, MeOH); UV (MeOH) *λ*_max_ (log *ε*) 276 (3.88), 201 (4.55) nm; ECD (MeOH) *λ* (Δ*ε*) 201 (−0.61), 215 (−3.80), 246 (+0.23); ^1^H and ^13^C NMR data, see Table 2; HRESIMS *m*/*z* 431.1339 [M − H]^−^ (calcd for C_22_H_23_O_9_, 431.1348).

Xuejiein G (**3**): White solids; $[\alpha {]}_{D}^{20}$ +56 (*c* 0.03, MeOH); UV (MeOH) *λ*_max_ (log *ε*) 277 (3.85), 204 (4.44) nm; ECD (MeOH) *λ* (Δ*ε*) 200 (+1.59), 220 (−2.15), 257 (+0.09), 283 (−0.08); ^1^H and ^13^C NMR data, see Table 2; HRESIMS *m*/*z* 431.1348 [M − H]^−^ (calcd for C_22_H_23_O_9_, 431.1348).

Xuejiein H (**4**): Yellowish gums; $[\alpha {]}_{D}^{20}$ +12 (*c* 0.02, MeOH); UV (MeOH) *λ*_max_ (log *ε*) 277 (3.36), 201 (4.23) nm; ECD (MeOH) *λ* (Δ*ε*) 200 (+2.05), 220 (−3.63), 251 (+0.23); ^1^H and ^13^C NMR data, see Table 3; HRESIMS *m*/*z* 431.1350 [M − H]^−^ (calcd for C_22_H_23_O_9_, 431.1348).

Xuejiein I (**5**): Red gums; $[\alpha {]}_{D}^{20}$ +5 (*c* 0.03, MeOH); UV (MeOH) *λ*_max_ (log *ε*) 273 (3.78), 206 (4.32) nm; ECD (MeOH) *λ* (Δ*ε*) 201 (+0.73), 206 (+2.24), 213 (−0.47), 220 (+0.33); ^1^H and ^13^C NMR data, see Table 3; HRESIMS *m*/*z* 449.1450 [M − H]^−^ (calcd for C_22_H_25_O_10_, 449.1453).

### 3.5. ECD and NMR Calculations

The details of ECD and NMR quantum chemical calculations are described in the Supplementary Materials.

### 3.6. Bioassays

The details of anti-neuroinflammatory, neuroprotective, and antidepressant potential are included in the Supplementary Materials.

## 4. Conclusions

In summary, phytochemical investigation of the *D. cochinchinens* is exudates led to the isolation of a pair of novel enantiomeric stilbenolignans, (−)- and (+)-dracaenolignans A (**1**), and four flavonoid derivatives, xuejieins F–I (**2**–**5**). Notably, compound **1** features a rare 6/6/5/6-fused carbon scaffold incorporating four contiguous stereogenic centers. Inspired by the chemical defense properties of species, biological evaluation demonstrated that (−)-**1** significantly alleviates LPS-induced neuroinflammation and depression-like behaviors, revealing a clear case of enantioselective bioactivity within this structural class. Collectively, these findings not only substantially broaden the known scaffold diversity of natural stilbenolignans, but also provide a promising chiral molecular framework for the rational development of novel antidepressant agents.

## Figures and Tables

**Table 1:** ^1^H (500 MHz) and ^13^C (125 MHz) NMR data of compound **1** in CD_3_OD (*δ*_H_ in ppm, *J* in Hz).

	1			1	
Position	*δ*_H_	*δ*_C_	Position	*δ*_H_	*δ*_C_
1		141.2	14	7.01, d (8.5)	130.4
2		113.7	1′		136.7
3		156.6	2′		145.5
4	6.13, d (2.3)	101.3	3′		139.8
5		156.5	4′		149.7
6	5.88, d (2.3)	107.5	5′	6.84, s	105.3
7	3.64, dd (7.5, 7.0)	54.9	6′		131.8
8	4.20, d (7.0)	58.4	7′	3.30, overlapped	51.9
9		138.0	8′	3.94, ddd (8.0, 8.0, 4.4)	70.4
10	7.01, d (8.5)	130.4	9′	2.86, d (16.0, 4.4)	30.8
11	6.76, d (8.5)	116.1		2.53, dd (16.0, 8.0)	
12		156.8	2′-OCH_3_	3.23, s	60.0
13	6.76, d (8.5)	116.1	4′-OCH_3_	3.89, s	56.0

**Table 2:** ^1^H NMR and ^13^C NMR data of **2** and **3** in CD_3_OD (*δ*_H_ in ppm, *J* in Hz).

	2			3	
Position	*δ*_H_	*δ*_C_	Position	*δ*_H_	*δ*_C_
1		104.0	1		104.1
2		159.4	2		159.1
3		104.1	3		104.3
4		153.0	4		153.1
5	5.95, s	93.0	5	5.97, s	93.0
6		159.9	6		159.8
7	2.86, m	20.7	7	2.84, m	20.7
8	3.10, m	39.3	8	3.04, m	39.3
9		202.2	9		202.2
1′		129.8	1′		129.8
2′	7.90, d (8.8)	132.1	2′	7.89, d (8.8)	132.2
3′	6.82, d (8.8)	116.3	3′	6.81, d (8.8)	116.5
4′		164.1	4′		164.6
5′	6.82, d (8.8)	116.3	5′	6.81, d (8.8)	116.5
6′	7.90, d (8.8)	132.1	6′	7.89, d (8.8)	132.2
1″	3.25, d (16.4)	35.4	1″	3.44, d (16.4)	33.7
	3.08, d (16.4)			2.93, d (16.4)	
2″		116.8	2″		120.8
3″	4.01, d (8.3)	81.3	3″	4.19, d (3.8)	82.9
4″	4.10, dd (8.3, 7.2)	76.2	4″	3.96, dd (6.3, 3.8)	78.8
5″	3.89, td (7.2, 3.4)	84.2	5″	4.05, ddd (6.3, 5.2, 3.0)	85.0
6″	3.73, dd (11.8, 3.4)	65.4	6″	3.77, dd (12.0, 3.0)	62.8
	3.66, dd (11.8, 7.2)			3.66, dd (12.0, 5.2)	
6-OCH_3_	3.69, s	56.1	6-OCH_3_	3.70, s	56.1

**Table 3:** ^1^H (500 MHz) and ^13^C (125 MHz) NMR data of compounds **4** and **5** in CD_3_OD (*δ*_H_ in ppm, *J* in Hz).

	4			5	
Position	*δ*_H_	*δ*_C_	Position	*δ*_H_	*δ*_C_
1		104.3	1		108.9
2		159.1	2		156.4
3		104.2	3		105.0
4		153.4	4		155.3
5	6.00, s	93.4	5	6.00, s	91.8
6		159.9	6		159.6
7	2.85, m	20.8	7	2.87, m	20.0
8	3.04, m	39.4	8	3.07, m	39.5
9		201.9	9		202.8
1′		129.8	1′		129.8
2′	7.90, d (8.8)	132.1	2′	7.90, d (8.8)	132.1
3′	6.82, d (8.8)	116.3	3′	6.82, d (8.8)	116.2
4′		164.1	4′		163.8
5′	6.82, d (8.8)	116.3	5′	6.82, d (8.8)	116.2
6′	7.90, d (8.8)	132.1	6′	7.90, d (8.8)	132.1
1″	3.23, d (16.6)	37.7	1″	5.12, d (1.2)	77.9
	2.95, d (16.6)				
2″		113.6	2″	3.99, dd (3.2, 1.2)	74.1
3″	3.84, d (2.8)	73.8	3″	3.63, dd (9.3, 3.2)	76.3
4″	3.86, dd (9.3, 2.8)	73.3	4″	3.75, dd (9.3, 9.3)	68.5
5″	3.67, m	67.4	5″	3.36, ddd (9.3, 5.5, 2.3)	83.2
6″	3.91, m	65.5	6″	3.91, dd (12.1. 2.3)	62.9
	3.66, m			3.78, dd (12.1, 5.5)	
6-OCH_3_	3.72, s	56.1	6-OCH_3_	3.69, s	55.7

## Data Availability

All data generated or analyzed in this study are provided within the article. Additional information is available from the corresponding author upon request.

## Supplementary Materials

The following supporting information can be downloaded at: https://www.mdpi.com/article/10.3390/ijms27093966/s1.
