# Supplementary material for: Skeletal Stilbenolignan Enantiomers and Flavonoid Derivatives Against Depression in Mice from Dracaena cochinchinensis Exudates
Source: Int J Mol Sci. 2026 Apr 29;27(9):3966. doi: 10.3390/ijms27093966 (PMC13163946; doi:10.3390/ijms27093966)
Supplement: Supplementary file 1 [file ijms-27-03966-s001.zip › ijms-4252858-supplementary.pdf]

Supplementary data for

Skeletal Stilbenolignan Enantiomers and Flavonoid Derivatives against Depression in Mice from *Dracaena cochinchinensis* Exudates

Tian-Chang He,[1,#] Fan Fang,[1,#] Wei-Fen Li,[1] Wen-Jing Yao,[1] Jing-Jing Qi,[2,*] and Yong-Xian Cheng[1,*]

[1] *Guangdong Provincial Key Laboratory of Chinese Medicine Ingredients and Gut Microbiomics. Institute for Inheritance-Based Innovation of Chinese Medicine, School of Pharmacy, Shenzhen University Medical School, Shenzhen 518060, PR China*

[2] *Jiangxi Province Key Laboratory of Natural and Biomimetic Drugs Research, College of Pharmacy, Jiangxi Normal University, Nanchang 330022, PR China*

[*]Corresponding authors.
E-mail addresses: yxcheng@szu.edu.cn (Y.-X. Cheng), qijing023@163.com (J.-J. Qi).

Table of Contents

1 Chiral Separation..........4

Figure S1. Chiral HPLC chromatogram of **1**..........4

2 HPLC Analysis of Compounds 1–5..........4

Figure S2. HPLC Analysis of chromatogram of (+)-**1**..........4

Figure S3. HPLC Analysis of chromatogram of (−)-**1**..........5

Figure S4. HPLC Analysis of chromatogram of **2**..........5

Figure S5. HPLC Analysis of chromatogram of **3**..........5

Figure S6. HPLC Analysis of chromatogram of **4**..........6

Figure S7. HPLC Analysis of chromatogram of **5**..........6

3 NMR, MS, Spectra of Compounds 1–5..........7

Figure S8. ^{1}H NMR spectrum of dracaenolignan A (**1**) in CD_3OD..........7

Figure S9. ^{13}C NMR and DEPT spectra of dracaenolignan A (**1**) in CD_3OD..........7

Figure S10. HSQC spectrum of dracaenolignan A (**1**) in CD_3OD..........8

Figure S11. HMBC spectrum of dracaenolignan A (**1**) in CD_3OD..........8

Figure S12. ^{1}H–^{1}H COSY spectrum of dracaenolignan A (**1**) in CD_3OD..........9

Figure S13. ROESY spectrum of dracaenolignan A (**1**) in CD_3OD..........9

Figure S14. (−)-HRESIMS of dracaenolignan A (**1**)..........10

Figure S15. ^{1}H NMR spectrum of xuejiein F (**2**) in CD_3OD..........11

Figure S16. ^{13}C NMR and DEPT spectra of xuejiein F (**2**) in CD_3OD..........11

Figure S17. HSQC spectrum of xuejiein F (**2**) in CD_3OD..........12

Figure S18. HMBC spectrum of xuejiein F (**2**) in CD_3OD..........12

Figure S19. ^{1}H–^{1}H COSY spectrum of xuejiein F (**2**) in CD_3OD..........13

Figure S20. ROESY spectrum of xuejiein F (**2**) in CD_3OD..........13

Figure S21. (−)-HRESIMS of xuejiein F (**2**)..........14

Figure S22. ^{1}H NMR spectrum of xuejiein G (**3**) in CD_3OD..........15

Figure S23. ^{13}C NMR and DEPT spectra of xuejiein G (**3**) in CD_3OD..........15

Figure S24. HSQC spectrum of xuejiein G (**3**) in CD_3OD..........16

Figure S25. HMBC spectrum of xuejiein G (**3**) in CD_3OD..........16

Figure S26. ^1H–^1H COSY spectrum of xuejiein G (**3**) in CD_3OD....17
Figure S27. ROESY spectrum of xuejiein G (**3**) in CD_3OD....17
Figure S28. (−)-HRESIMS of xuejiein G (**3**)....18
Figure S29. ^1H NMR spectrum of xuejiein H (**4**) in CD_3OD. 19
Figure S30. ^{13}C NMR and DEPT spectra of xuejiein H (**4**) in CD_3OD. 19
Figure S31. HSQC spectrum of xuejiein H (4) in CD_3OD. 20
Figure S32. HMBC spectrum of xuejiein H (**4**) in CD_3OD. 20
Figure S33. ^1H–^1H COSY spectrum of xuejiein H (**4**) in CD_3OD....21
Figure S34. ROESY spectrum of xuejiein H (**4**) in CD_3OD....21
Figure S35. (−)-HRESIMS of xuejiein H (**4**)....22
Figure S36. ^1H NMR spectrum of xuejiein I (**5**) in CD_3OD. 23
Figure S37. ^{13}C NMR and DEPT spectrum of xuejiein I (**5**) in CD_3OD. 23
Figure S38. HSQC spectrum of xuejiein I (**5**) in CD_3OD....24
Figure S39. HMBC spectrum of xuejiein I (**5**) in CD_3OD....24
Figure S40. ^1H–^1H COSY spectrum of xuejiein I (**5**) in CD_3OD. 25
Figure S41. ROESY spectrum of xuejiein I (**5**) in CD_3OD. 25
Figure S42. (−)-HRESIMS of xuejiein I (**5**). 26
4 Computational Calculations 27
4.1 Conformational Search Geometry Optimization and Frequency Calculations.27
4.2 NMR and DP4+ Calculations....27
4.3 ECD Calculations....28
Figure S43 Experimental and calculated chemical shifts of **1** and its possible isomers.29
Figure S44. Detailed DP4+ probabilities of all isomers for compound **1**....30
5 Bioassays 31
5.1 Cell Culture and Viability Assay 31
5.2 In Vivo Experiments 31
Figure S45 The cell viability of BV2 cells treated with various compound concentrations was measured using the CCK-8 assay, with three replicate wells per concentration. 32

1 Chiral Separation

The enantiomerically mixture (±)-**1** was subjected to a chiral HPLC column to afford the optical pure enantiomers (−)-**1** and (+)-**1**.

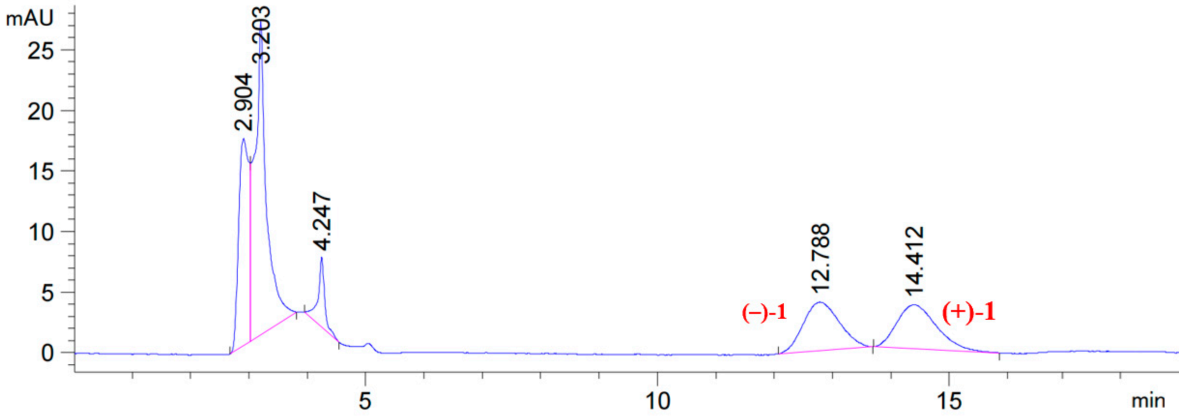

Figure S1. Chiral HPLC chromatogram of **1**.

2 HPLC Analysis of Compounds 1–5

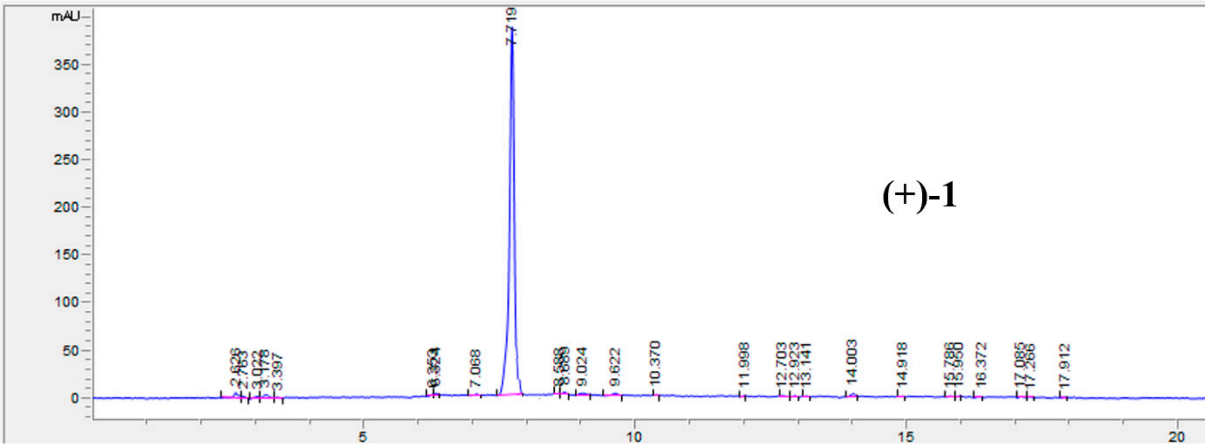

Figure S2. HPLC Analysis of chromatogram of (+)-**1**.

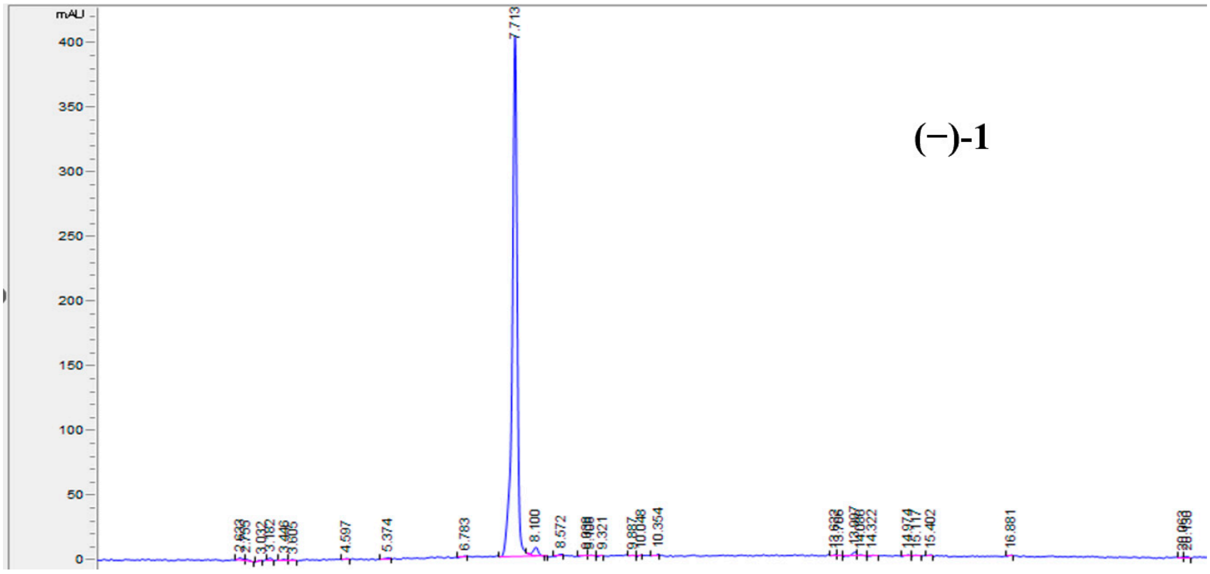

Figure S3. HPLC Analysis of chromatogram of (−)-**1**.

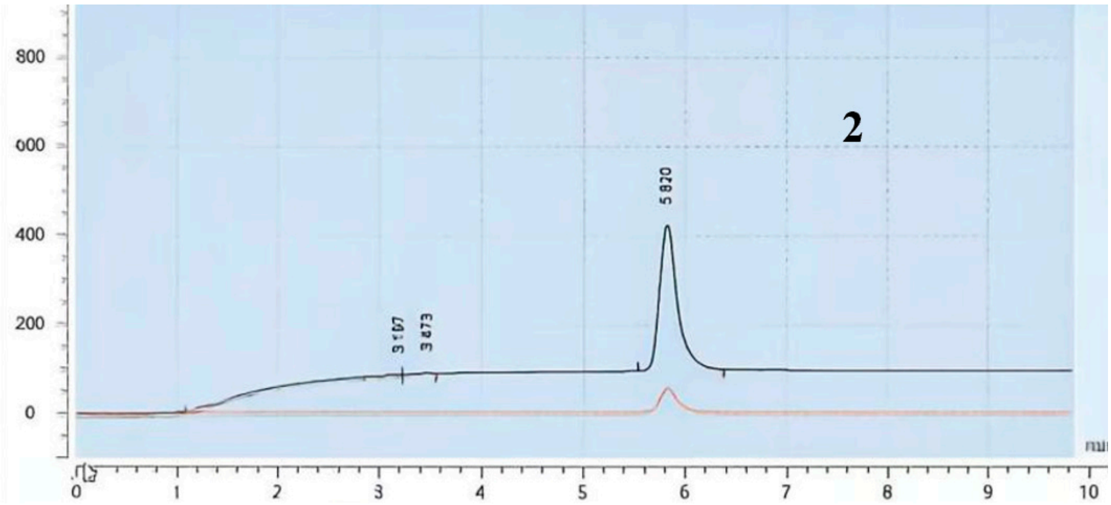

Figure S4. HPLC Analysis of chromatogram of **2**.

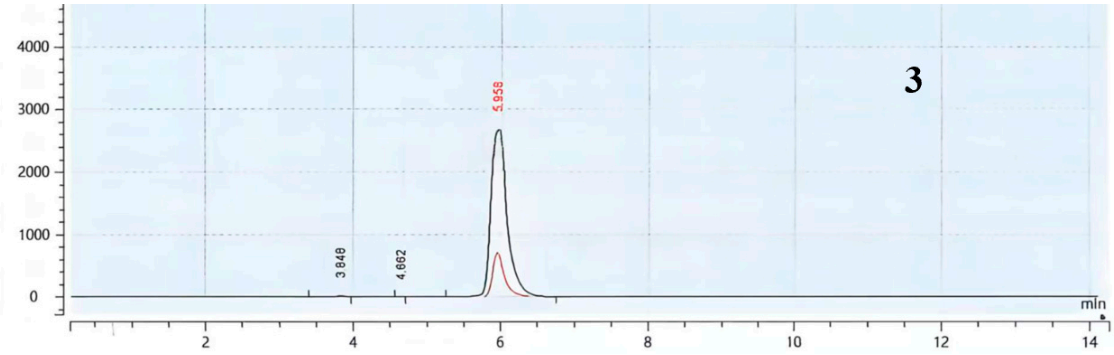

Figure S5. HPLC Analysis of chromatogram of **3**.

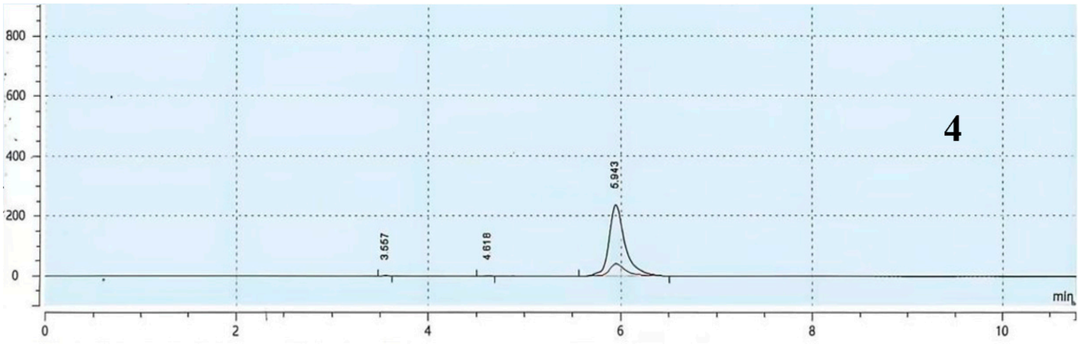

Figure S6. HPLC Analysis of chromatogram of **4**.

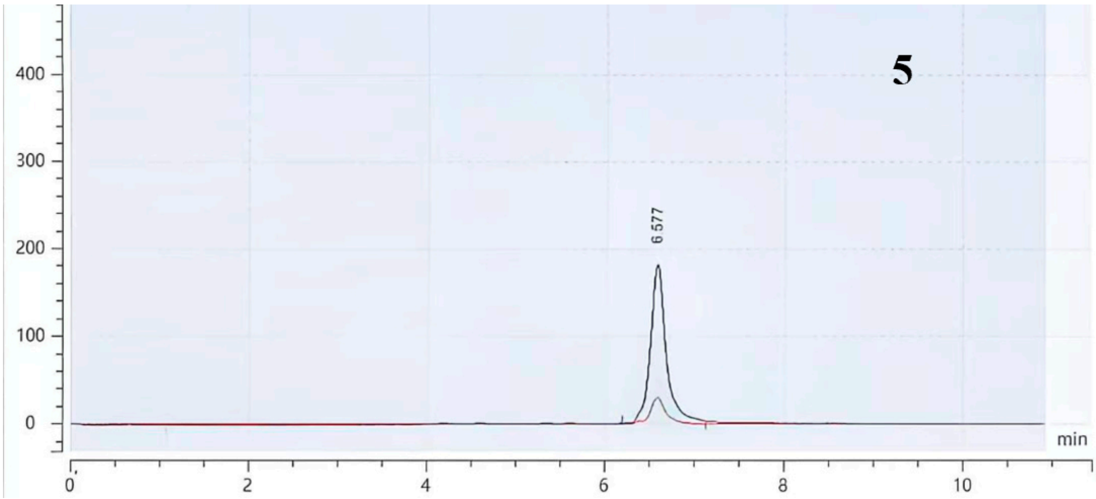

Figure S7. HPLC Analysis of chromatogram of **5**.

3 NMR, MS, Spectra of Compounds 1–5

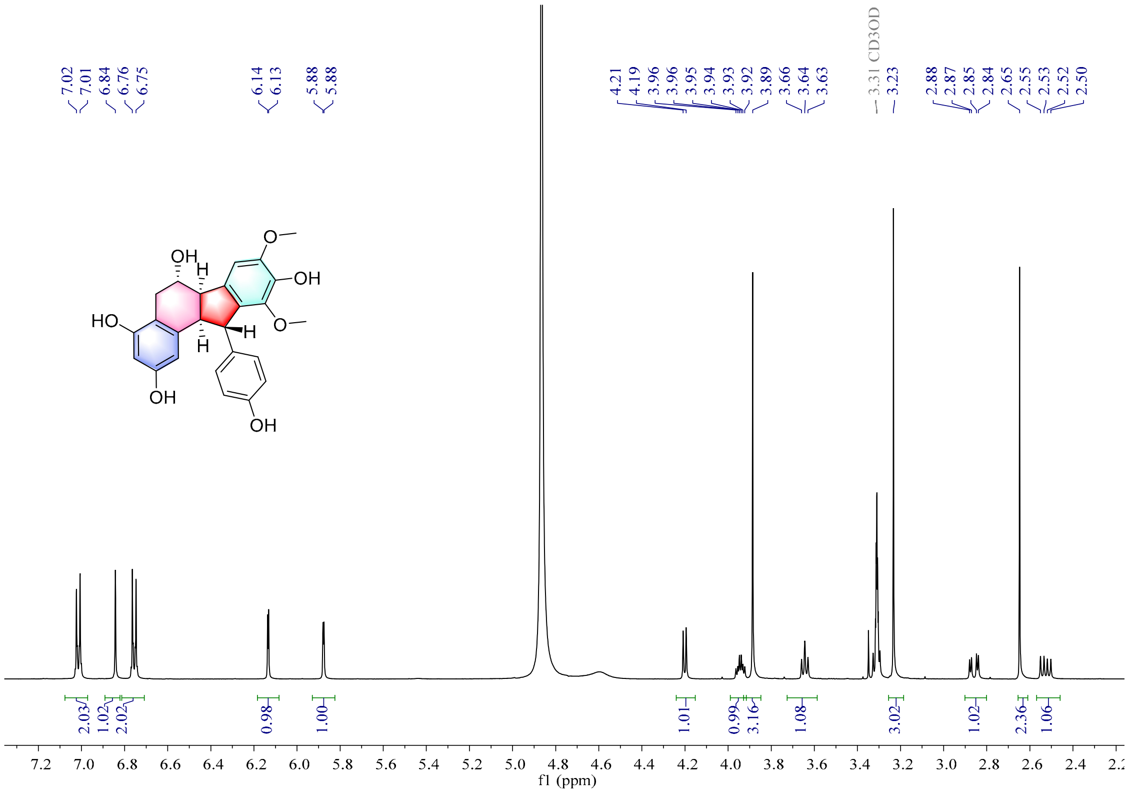

Figure S8. ^{1}H NMR spectrum of dracaenolignan A (**1**) in CD_3OD.

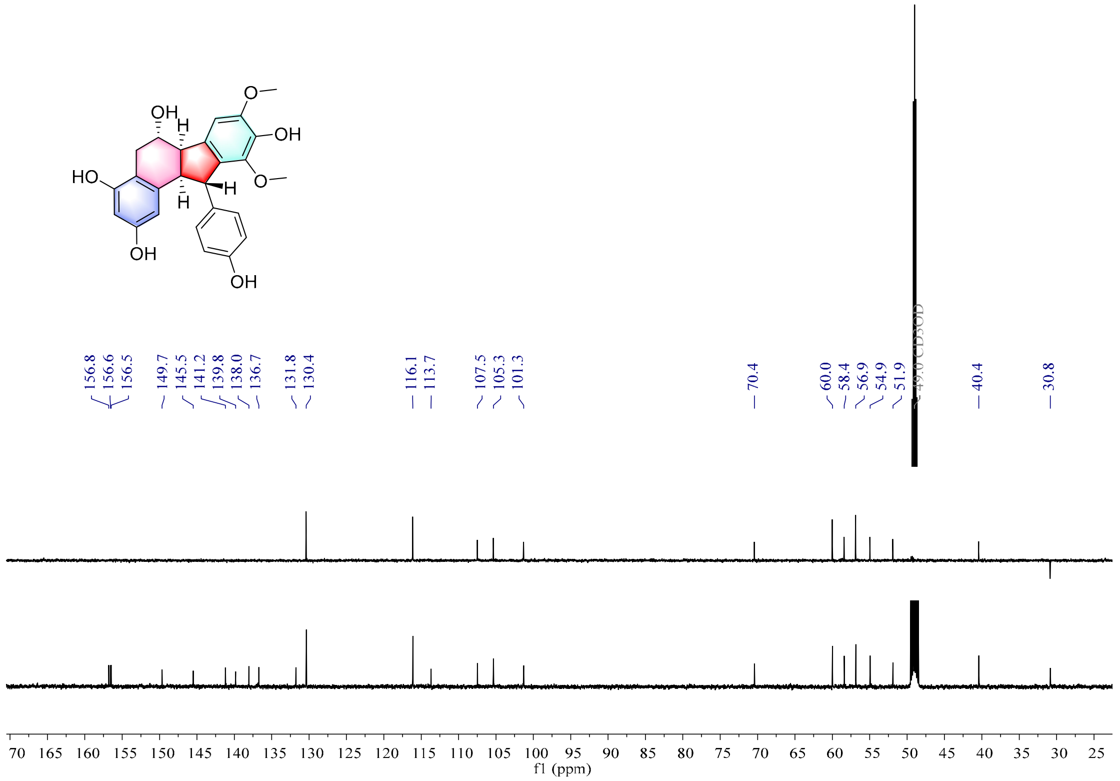

Figure S9. ^{13}C NMR and DEPT spectra of dracaenolignan A (**1**) in CD_3OD.

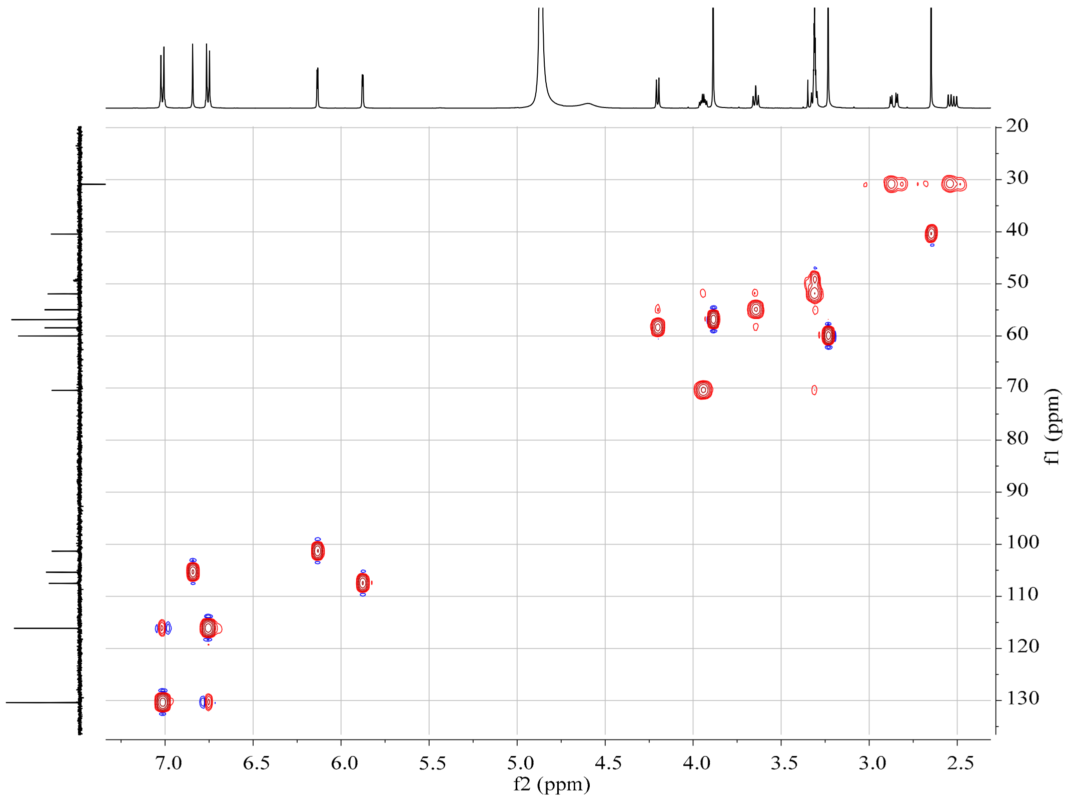

Figure S10. HSQC spectrum of dracaenolignan A (**1**) in CD_3OD.

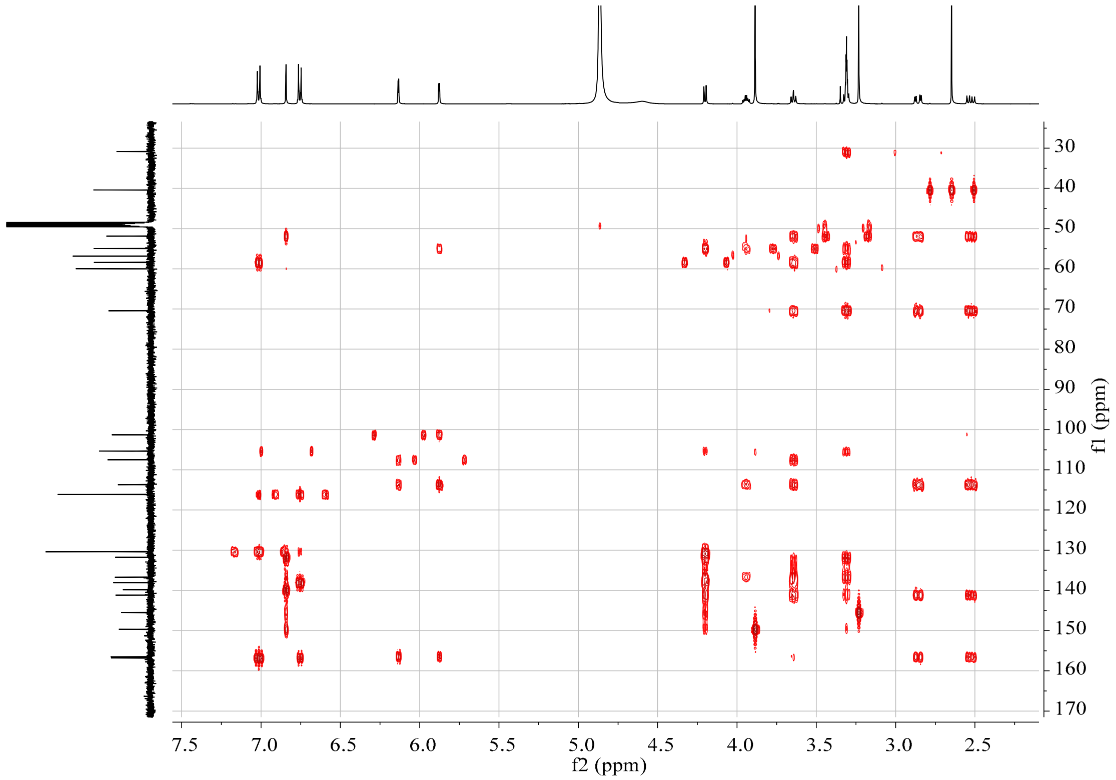

Figure S11. HMBC spectrum of dracaenolignan A (**1**) in CD_3OD.

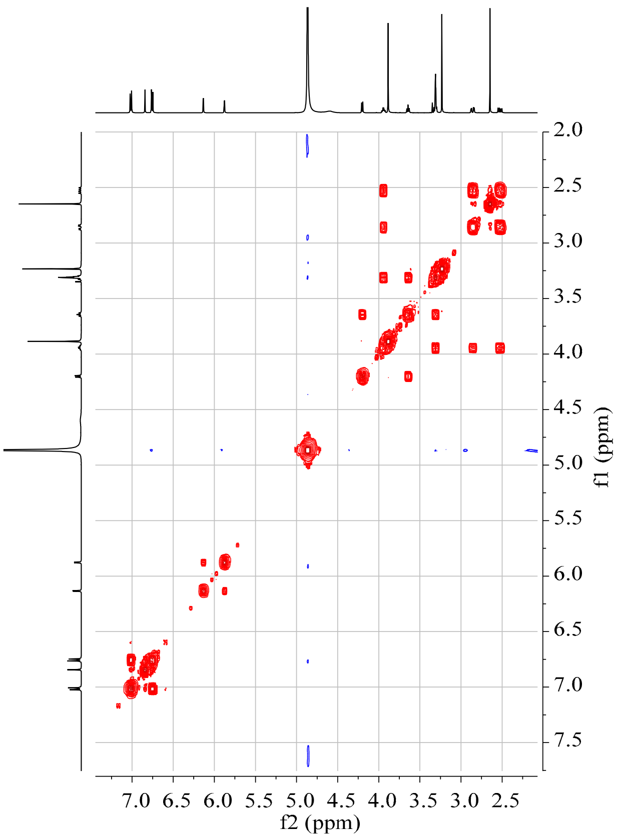

Figure S12. ^{1}H–^{1}H COSY spectrum of dracaenolignan A (**1**) in CD_3OD.

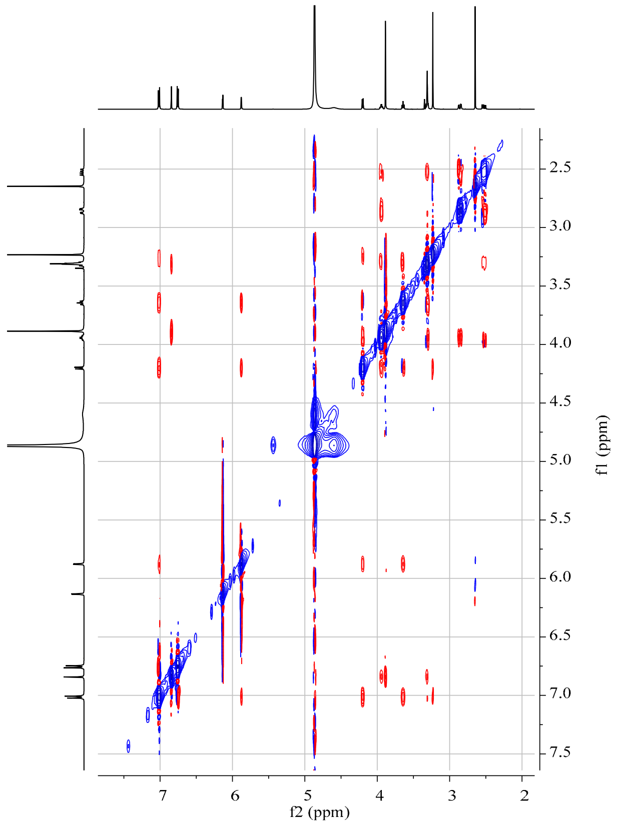

Figure S13. ROESY spectrum of dracaenolignan A (**1**) in CD_3OD.

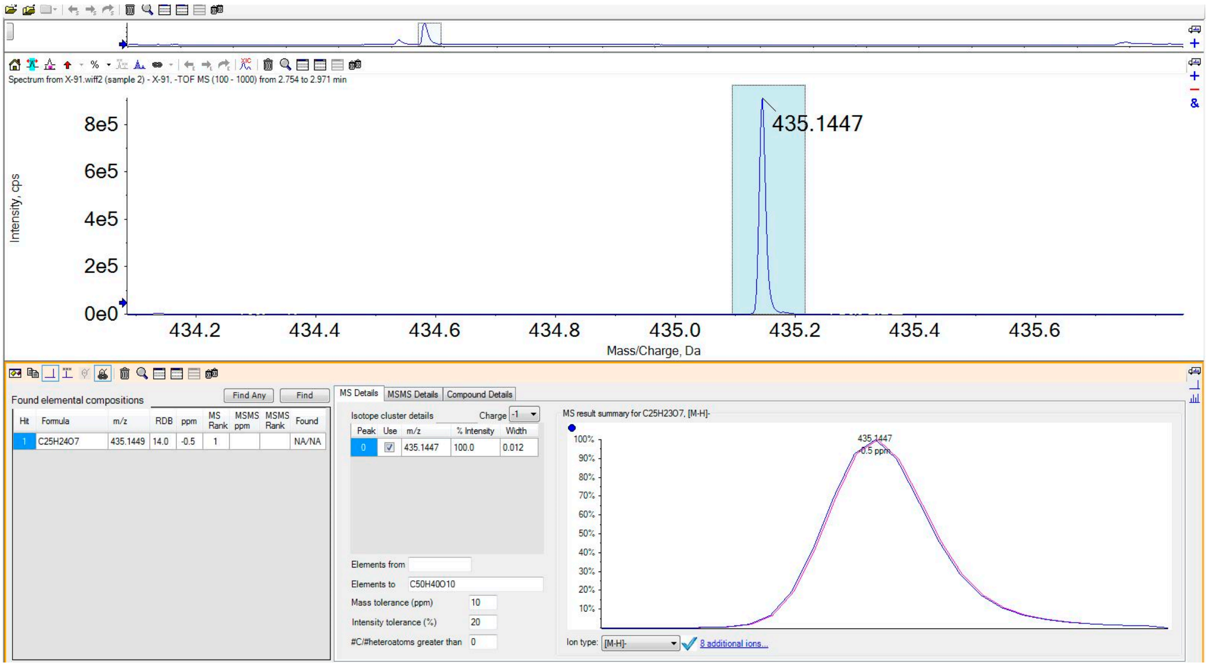

Figure S14. (−)-HRESIMS of dracaenolignan A (**1**).

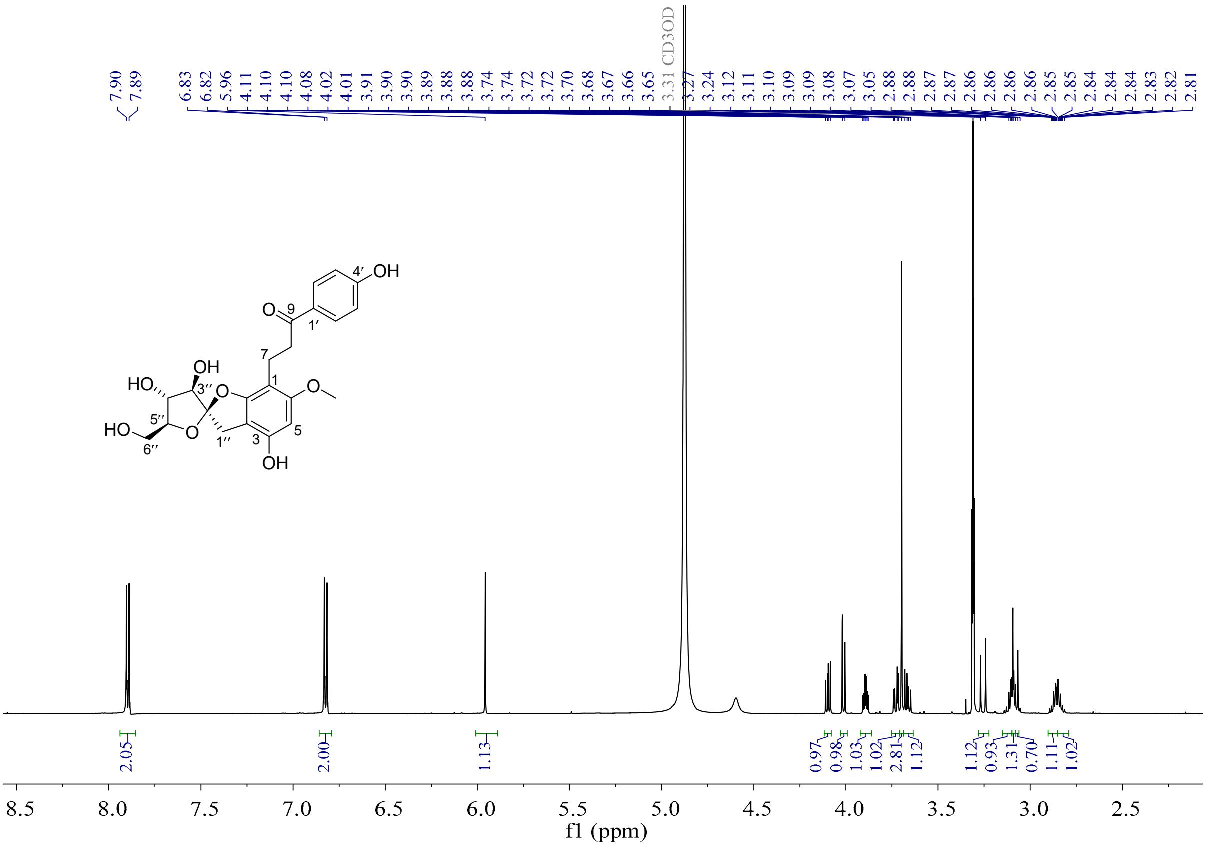

Figure S15. ^{1}H NMR spectrum of xuejiein F (**2**) in CD_3OD.

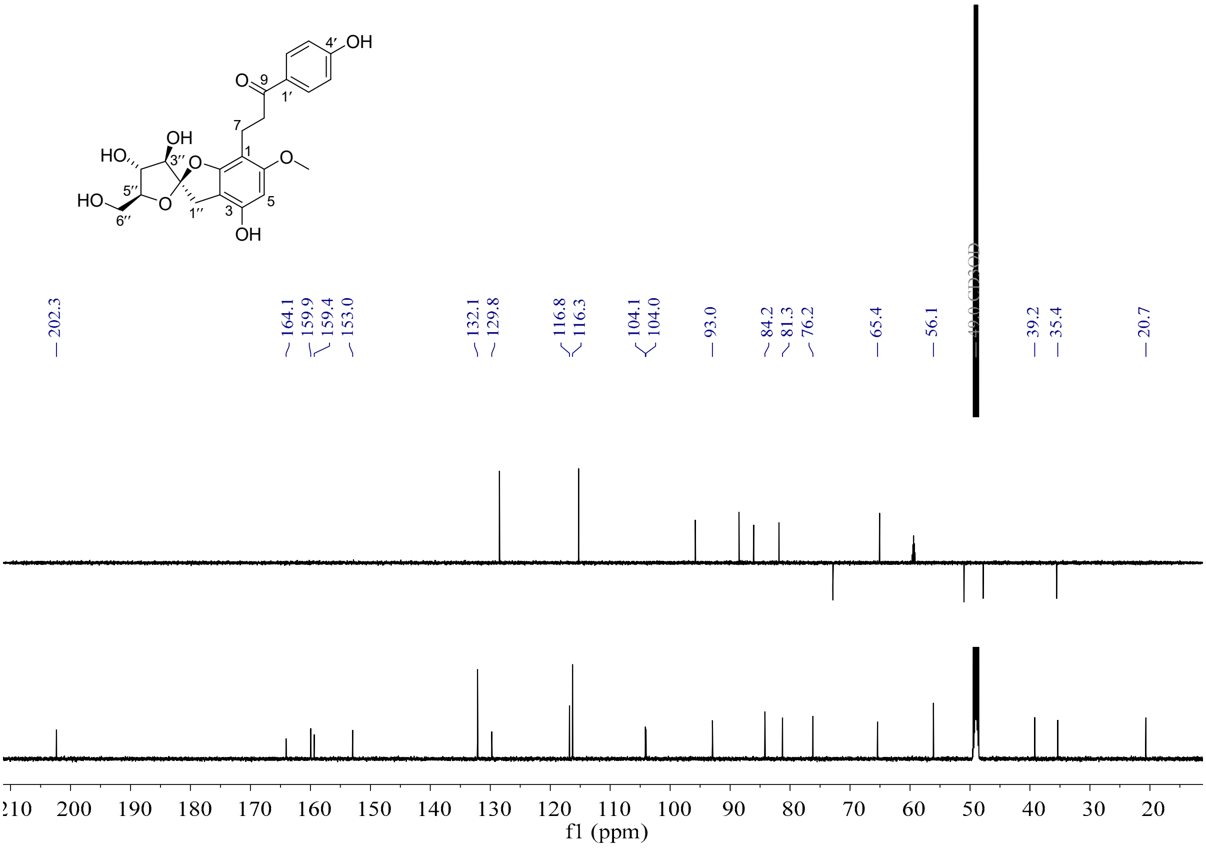

Figure S16. ^{13}C NMR and DEPT spectra of xuejiein F (**2**) in CD_3OD.

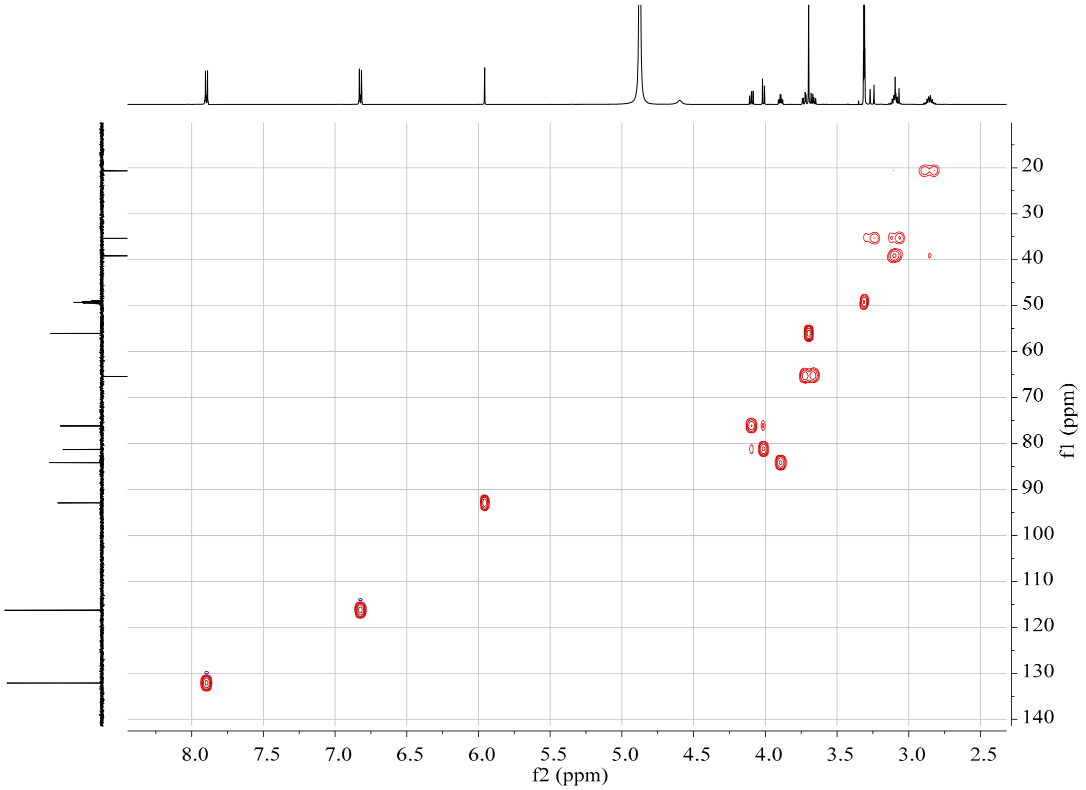

Figure S17. HSQC spectrum of xuejiein F (**2**) in CD3OD.

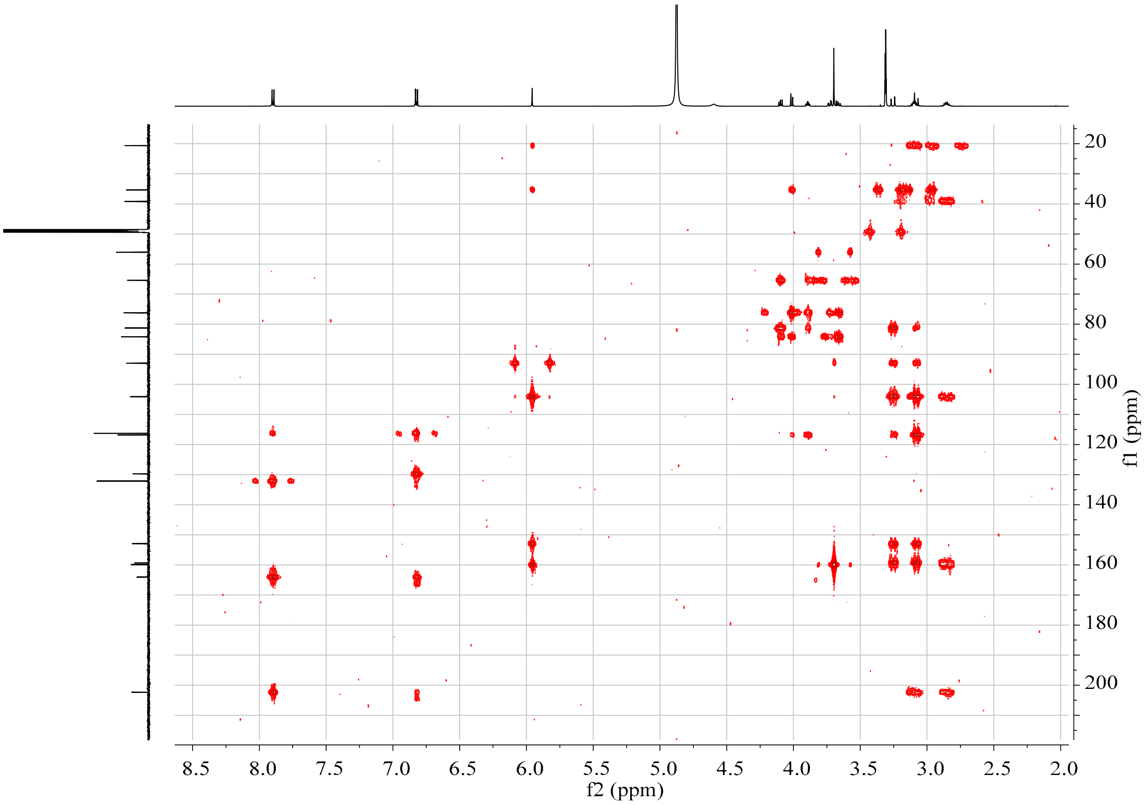

Figure S18. HMBC spectrum of xuejiein F (**2**) in CD3OD.

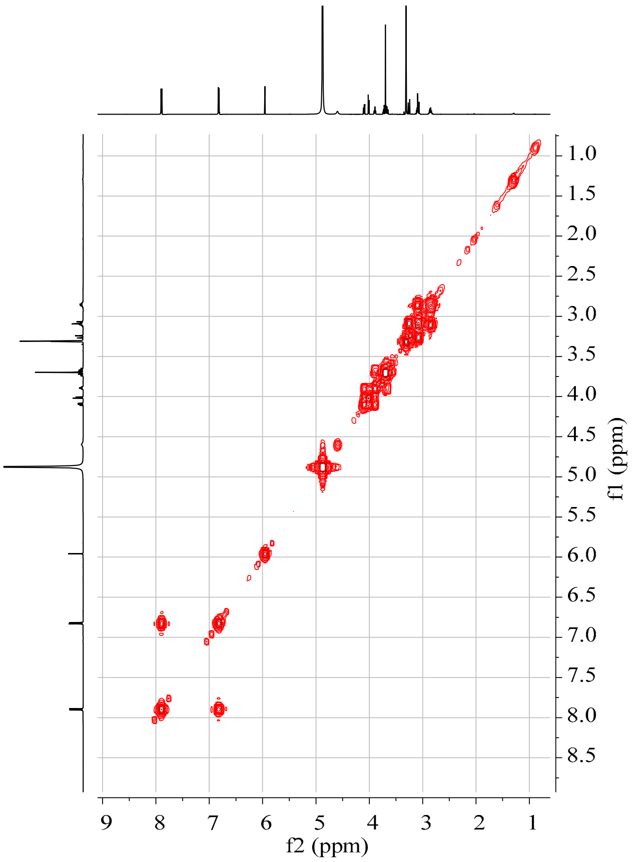

Figure S19. ^{1}H–^{1}H COSY spectrum of xuejiein F (**2**) in CD_3OD.

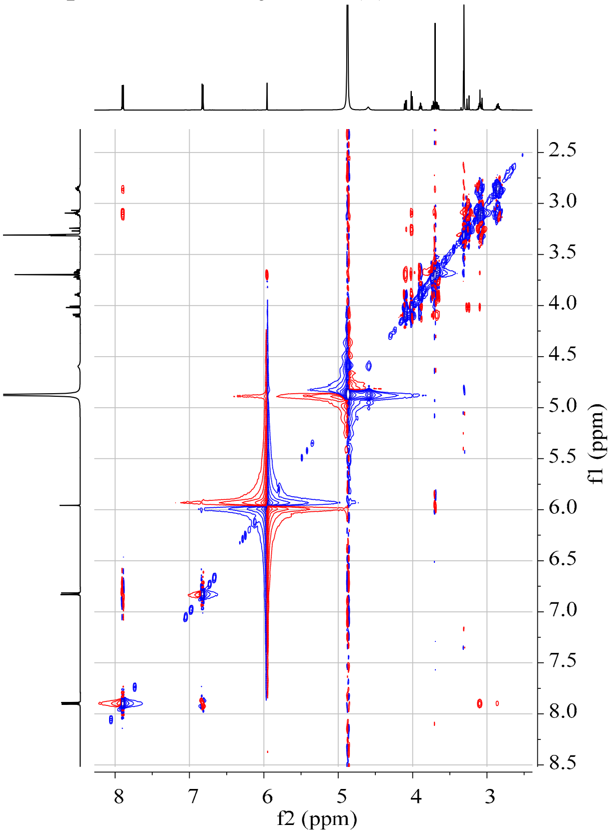

Figure S20. ROESY spectrum of xuejiein F (**2**) in CD_3OD.

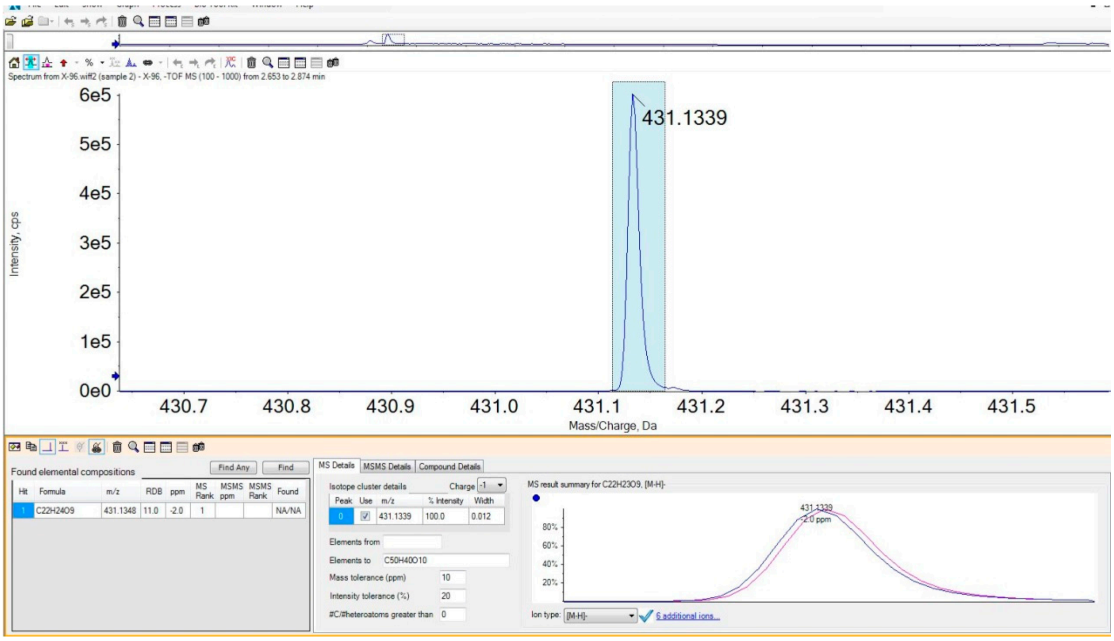

Figure S21. (−)-HRESIMS of xuejiein F (**2**).

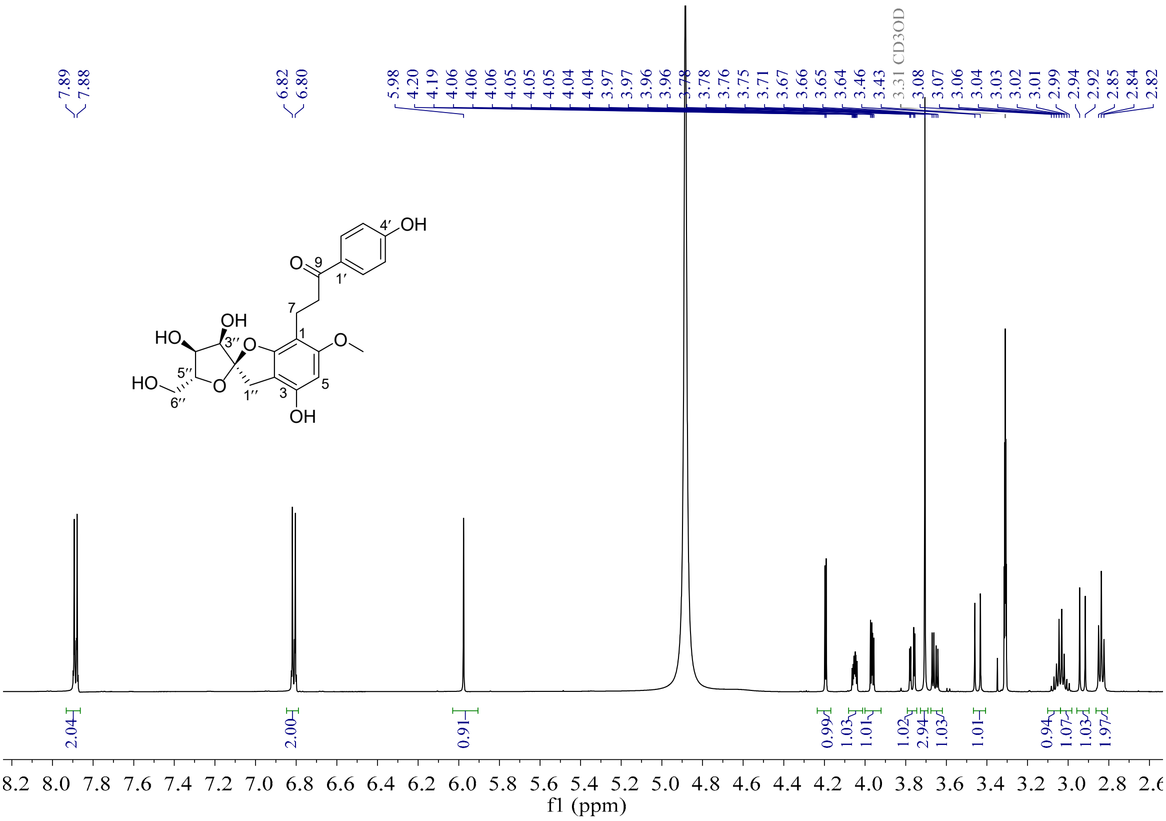

Figure S22. ^{1}H NMR spectrum of xuejiein G (**3**) in CD_3OD.

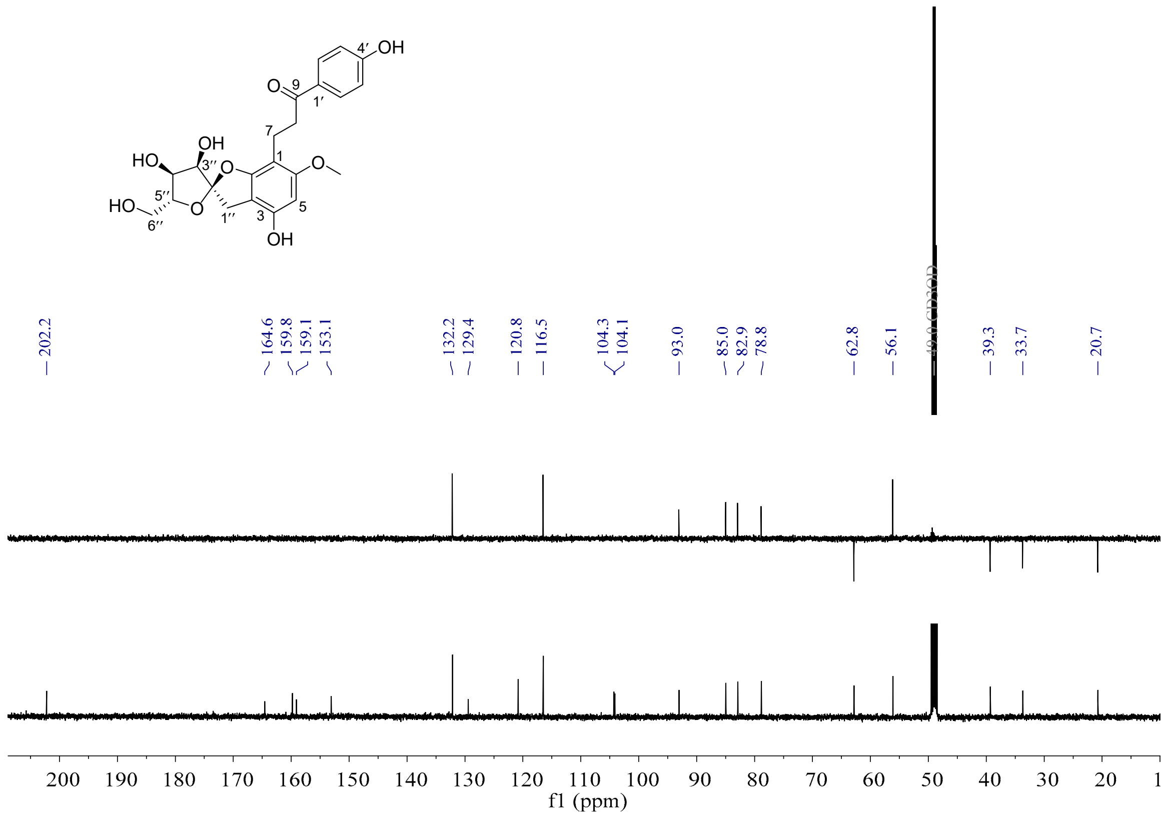

Figure S23. ^{13}C NMR and DEPT spectra of xuejiein G (**3**) in CD_3OD.

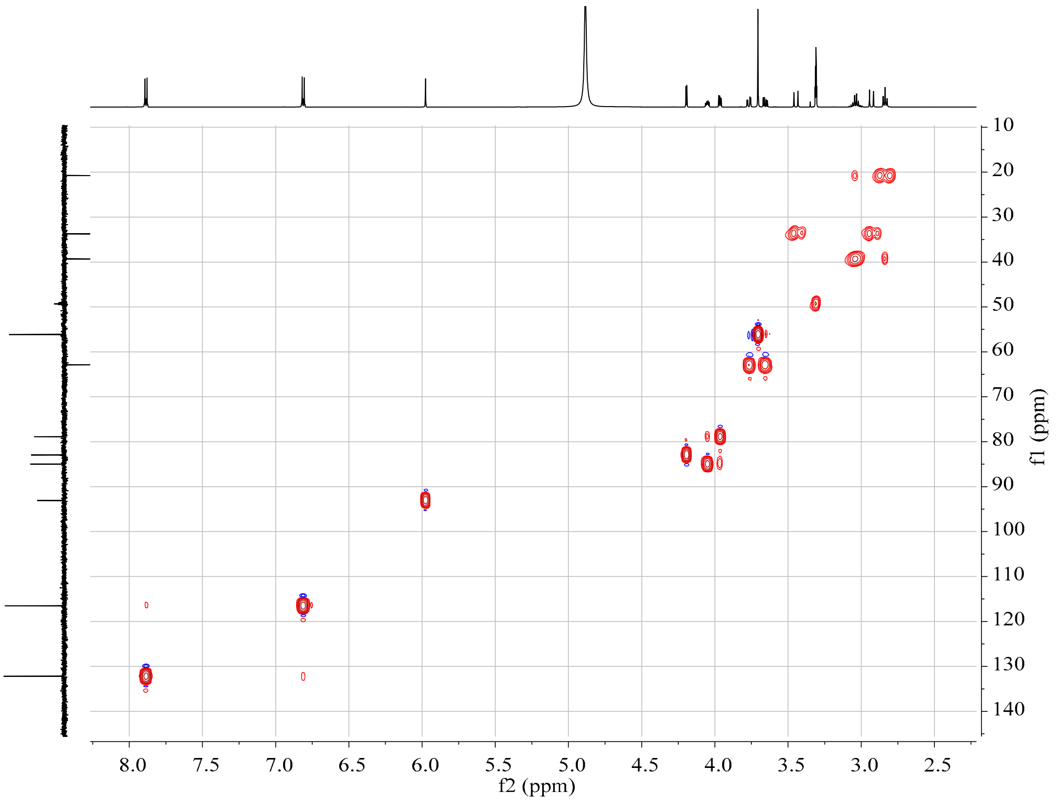

Figure S24. HSQC spectrum of xuejiein G (**3**) in CD_3OD.

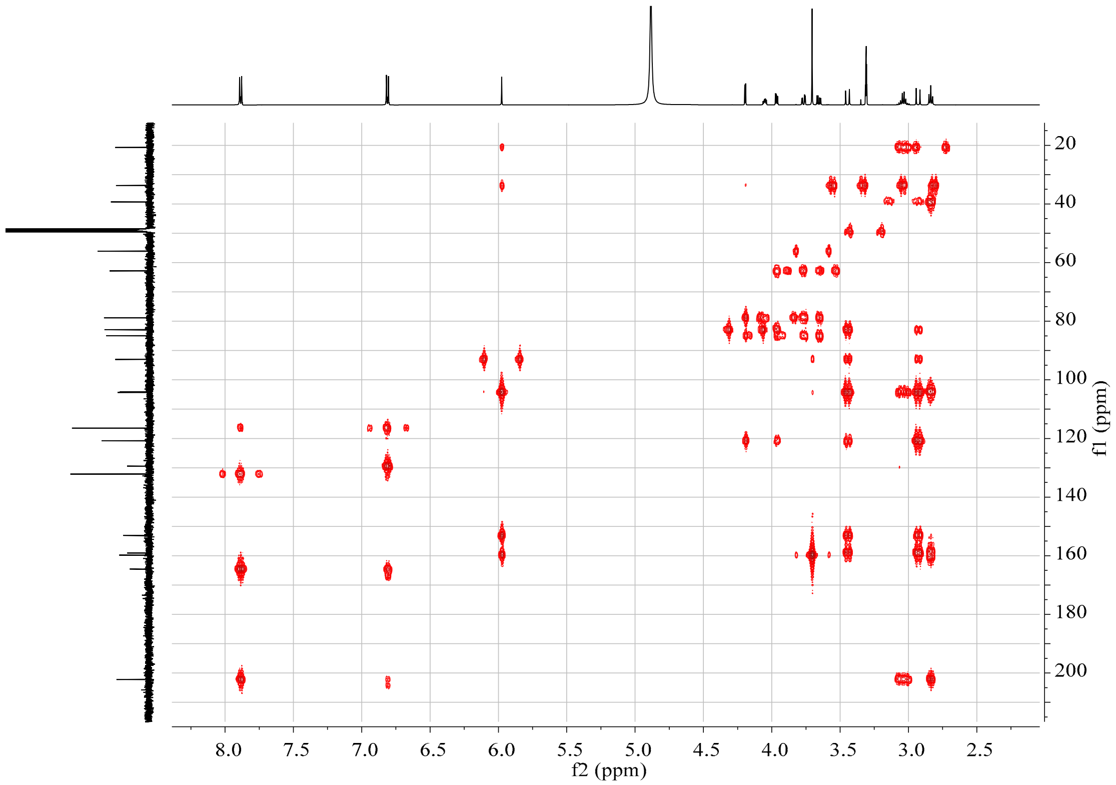

Figure S25. HMBC spectrum of xuejiein G (**3**) in CD_3OD.

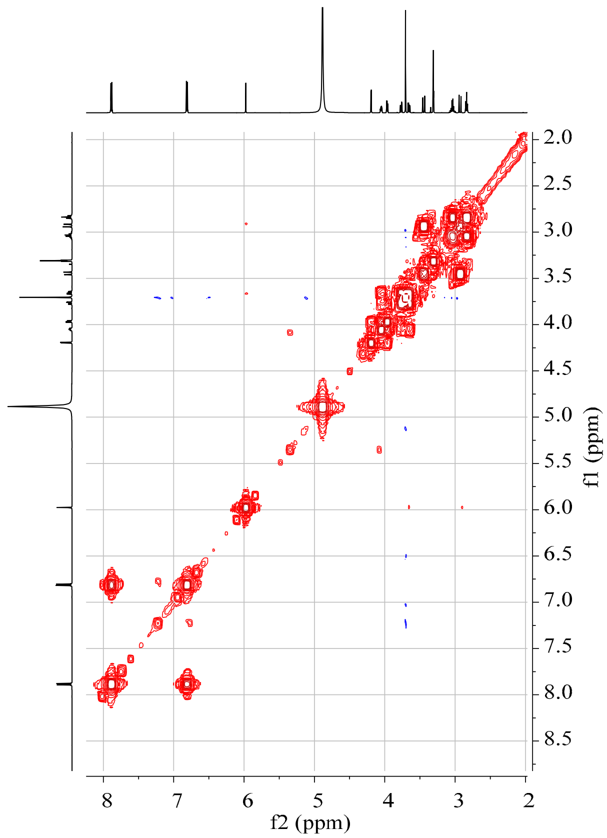

Figure S26. ^1H–^1H COSY spectrum of xuejiein G (**3**) in CD_3OD.

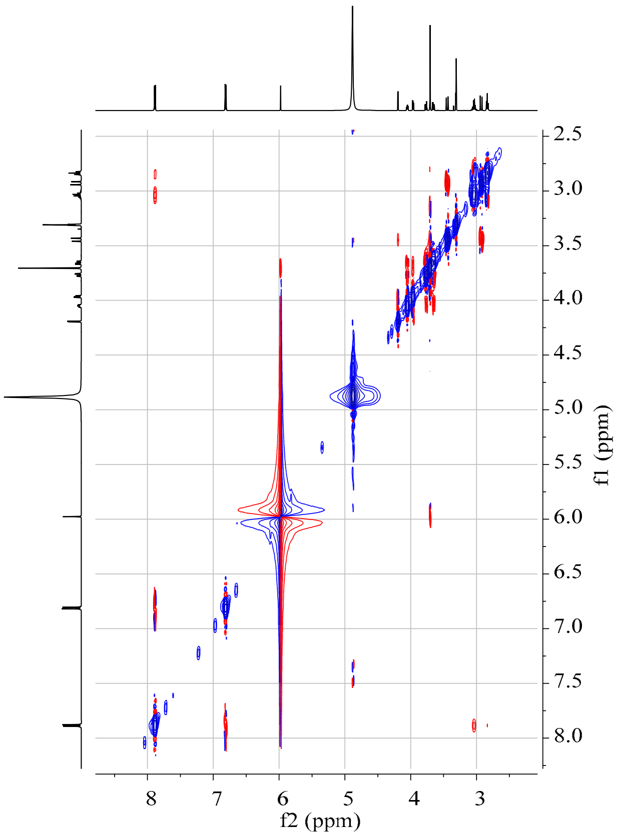

Figure S27. ROESY spectrum of xuejiein G (**3**) in CD_3OD.

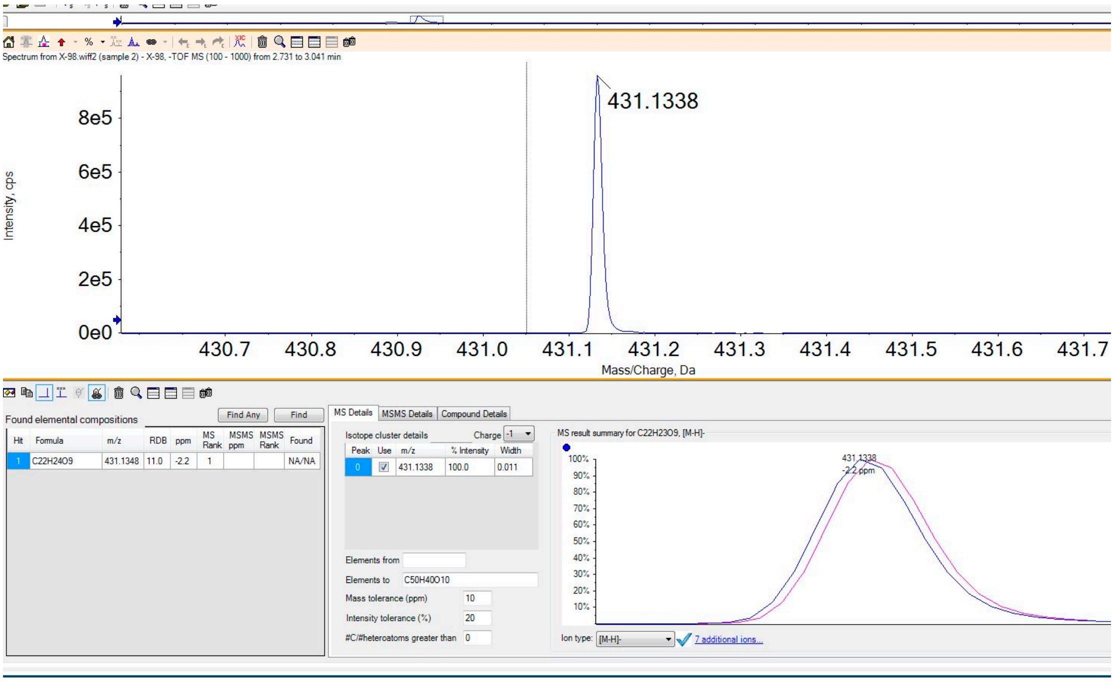

Figure S28. (−)-HRESIMS of xuejiein G (**3**).

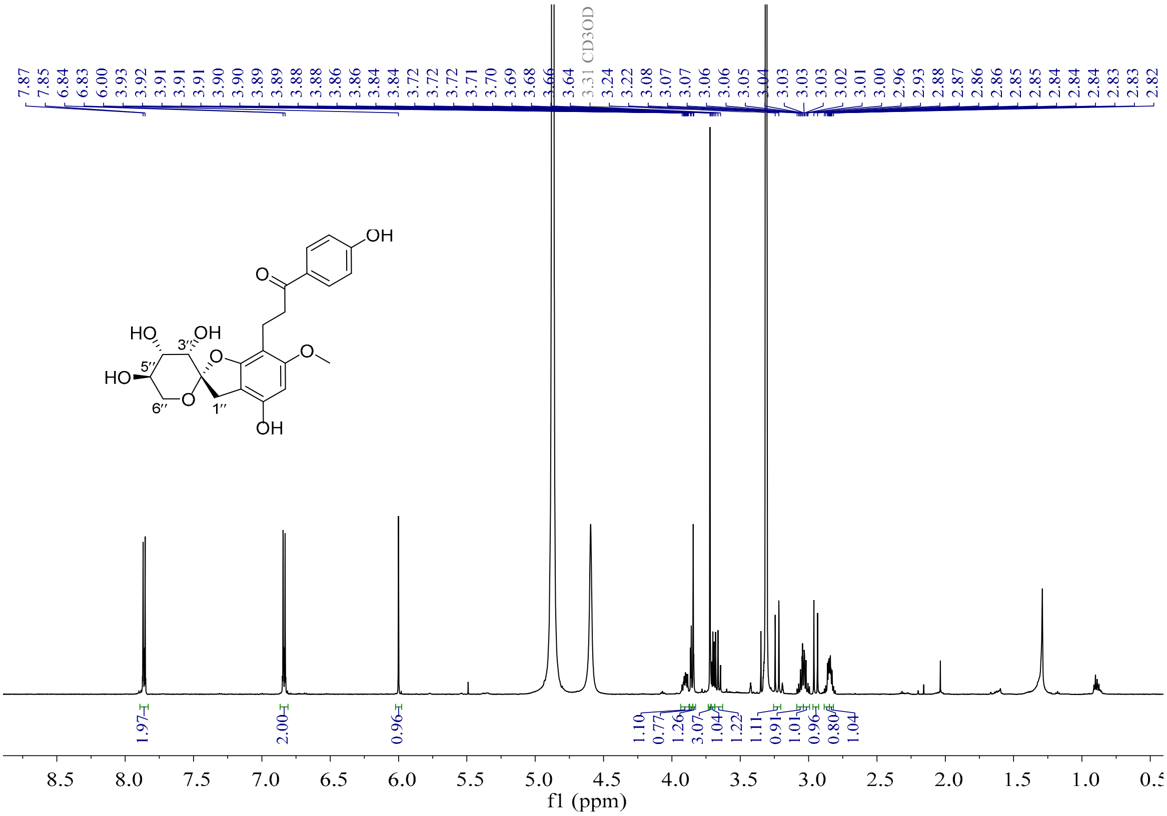

Figure S29. ^{1}H NMR spectrum of xuejiein H (**4**) in CD_3OD.

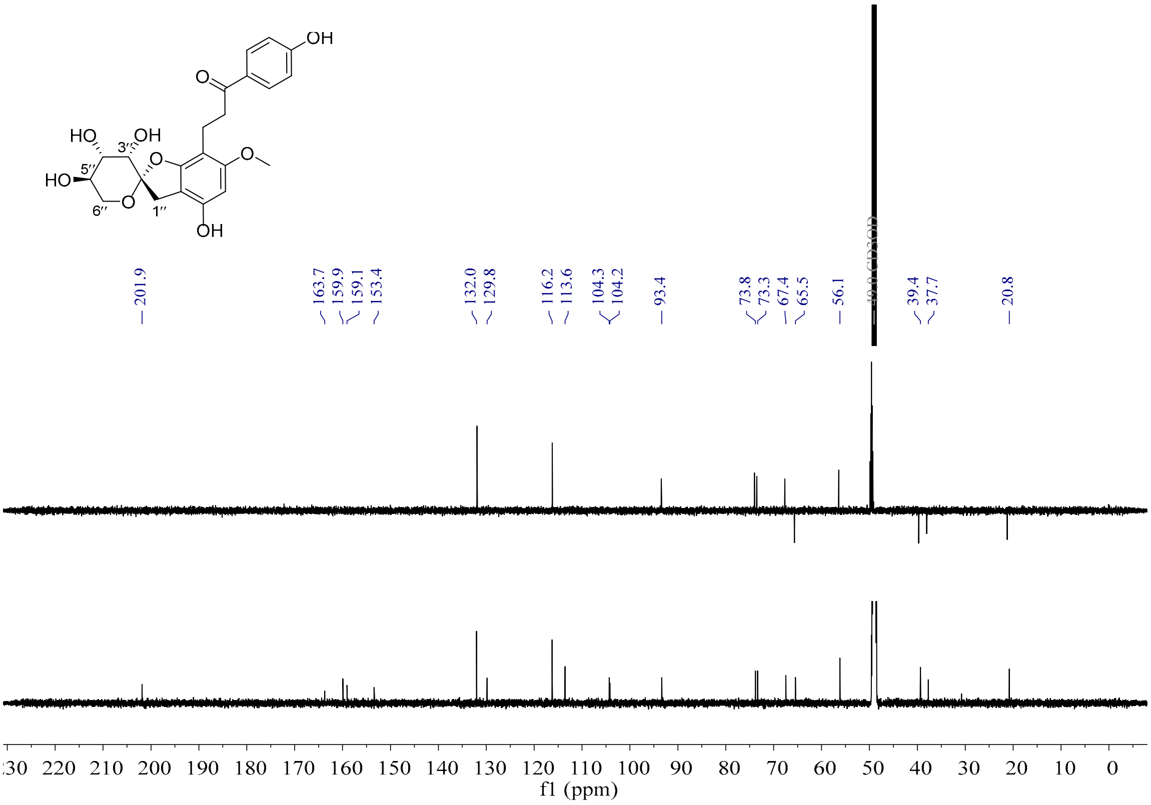

Figure S30. ^{13}C NMR and DEPT spectra of xuejiein H (**4**) in CD_3OD.

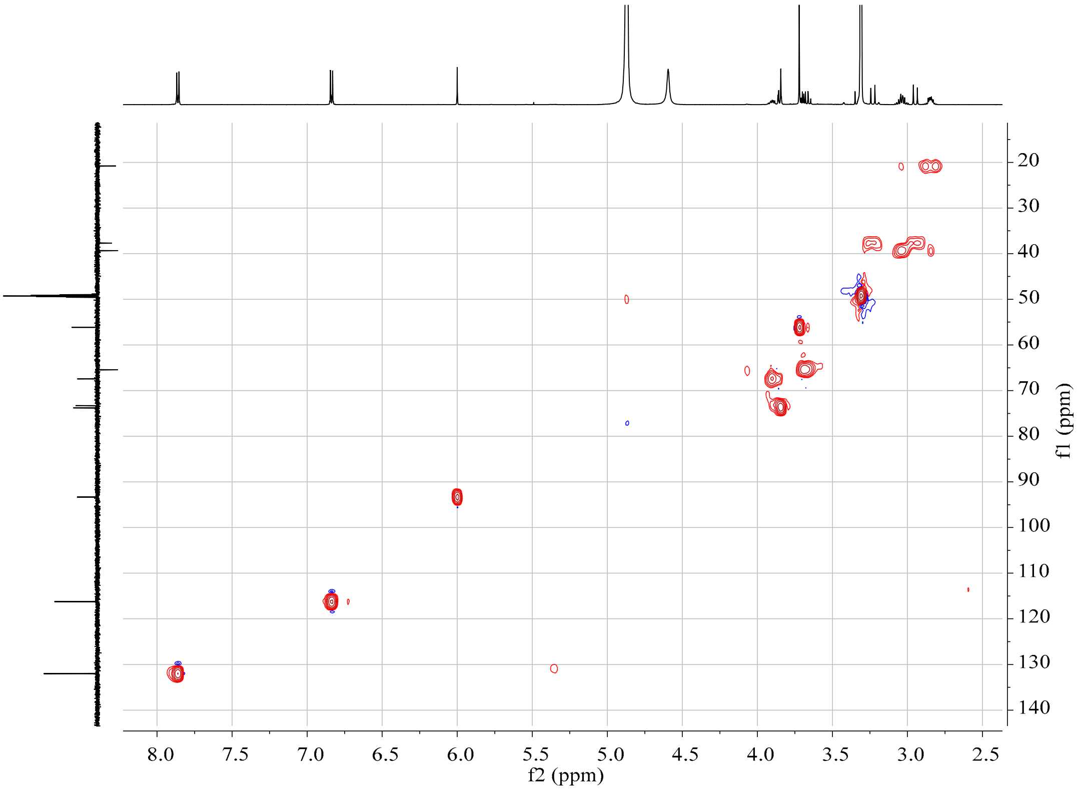

Figure S31. HSQC spectrum of xuejiein H (4) in CD_3OD.

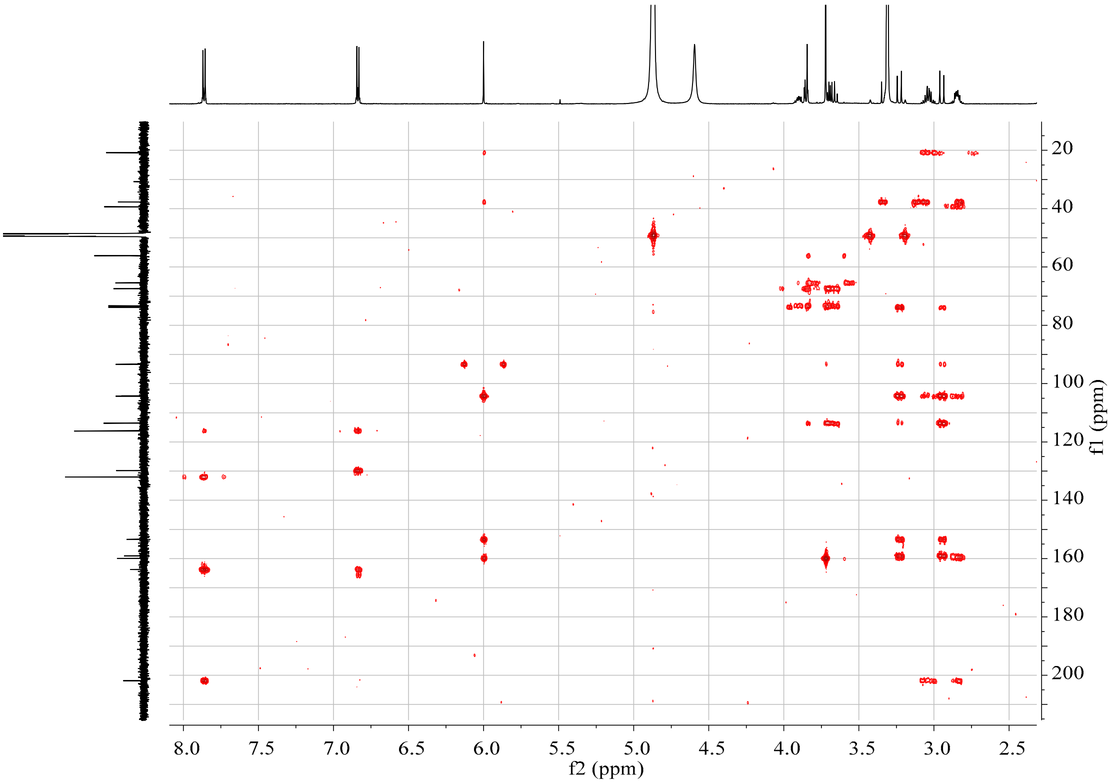

Figure S32. HMBC spectrum of xuejiein H (**4**) in CD_3OD.

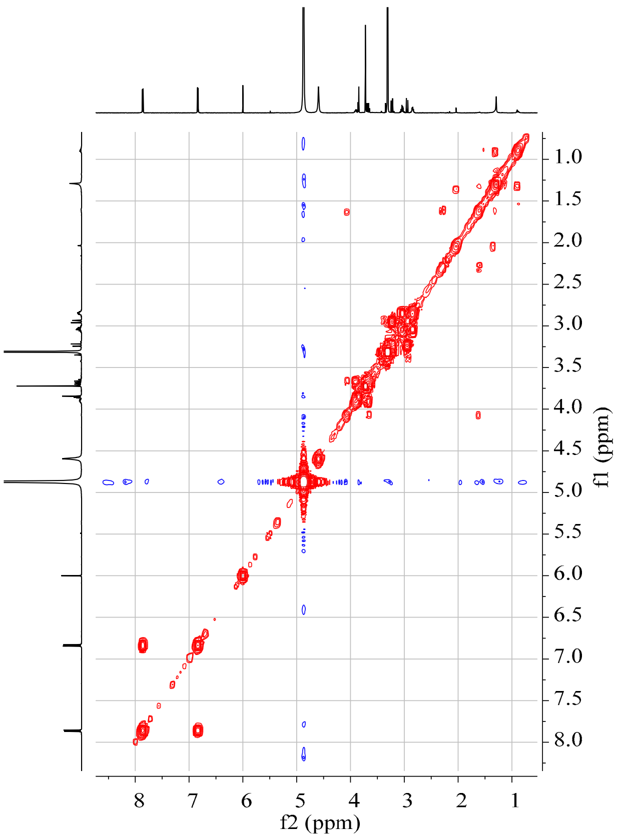

Figure S33. ^{1}H–^{1}H COSY spectrum of xuejiein H (**4**) in CD_3OD.

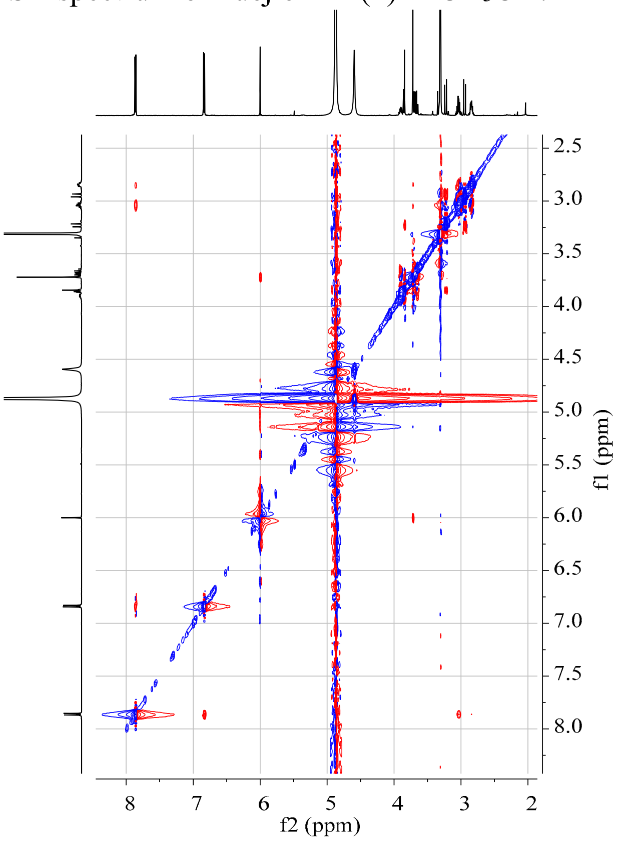

Figure S34. ROESY spectrum of xuejiein H (**4**) in CD_3OD.

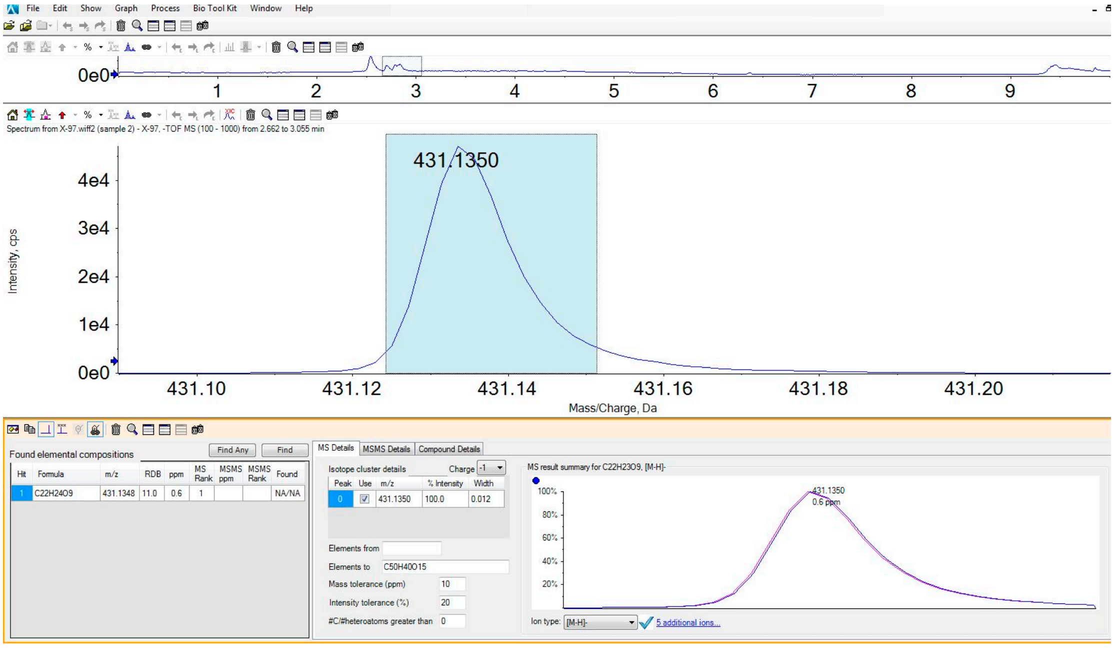

Figure S35. (−)-HRESIMS of xuejiein H (**4**).

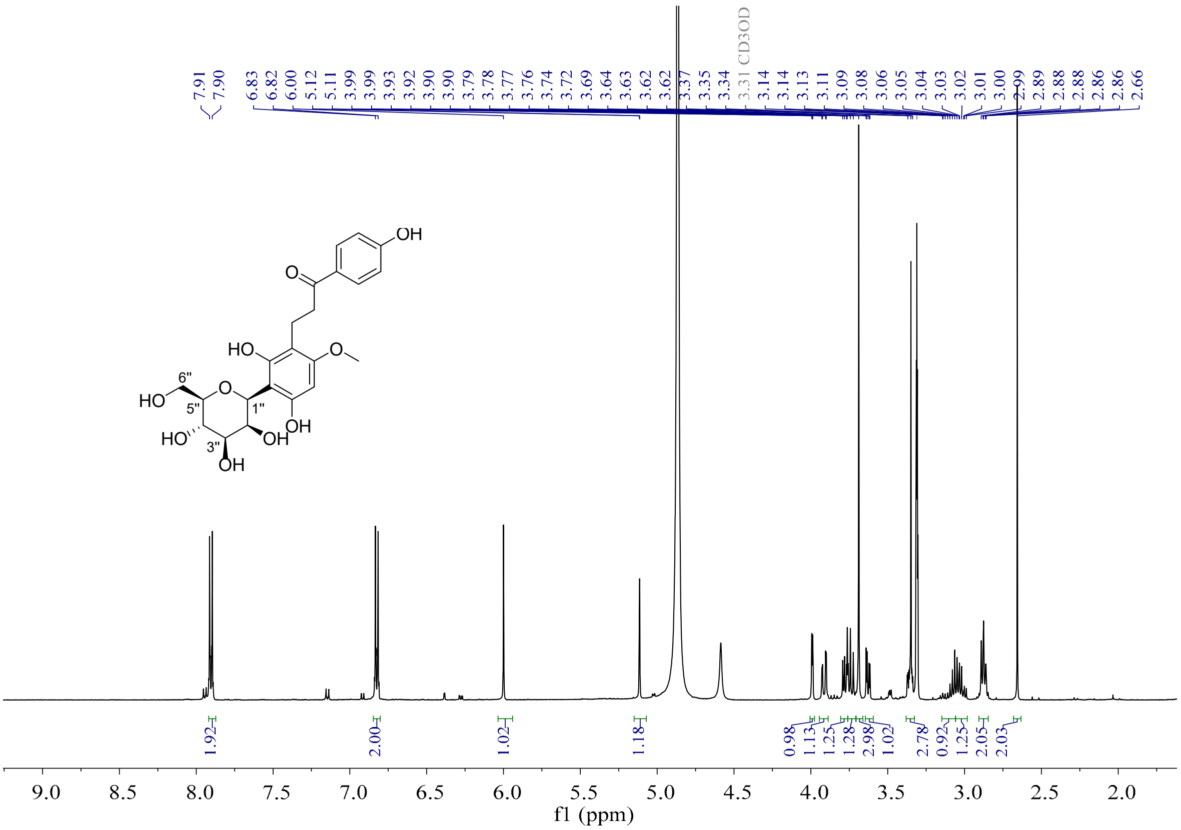

Figure S36. ^{1}H NMR spectrum of xuejiein I (**5**) in CD3OD.

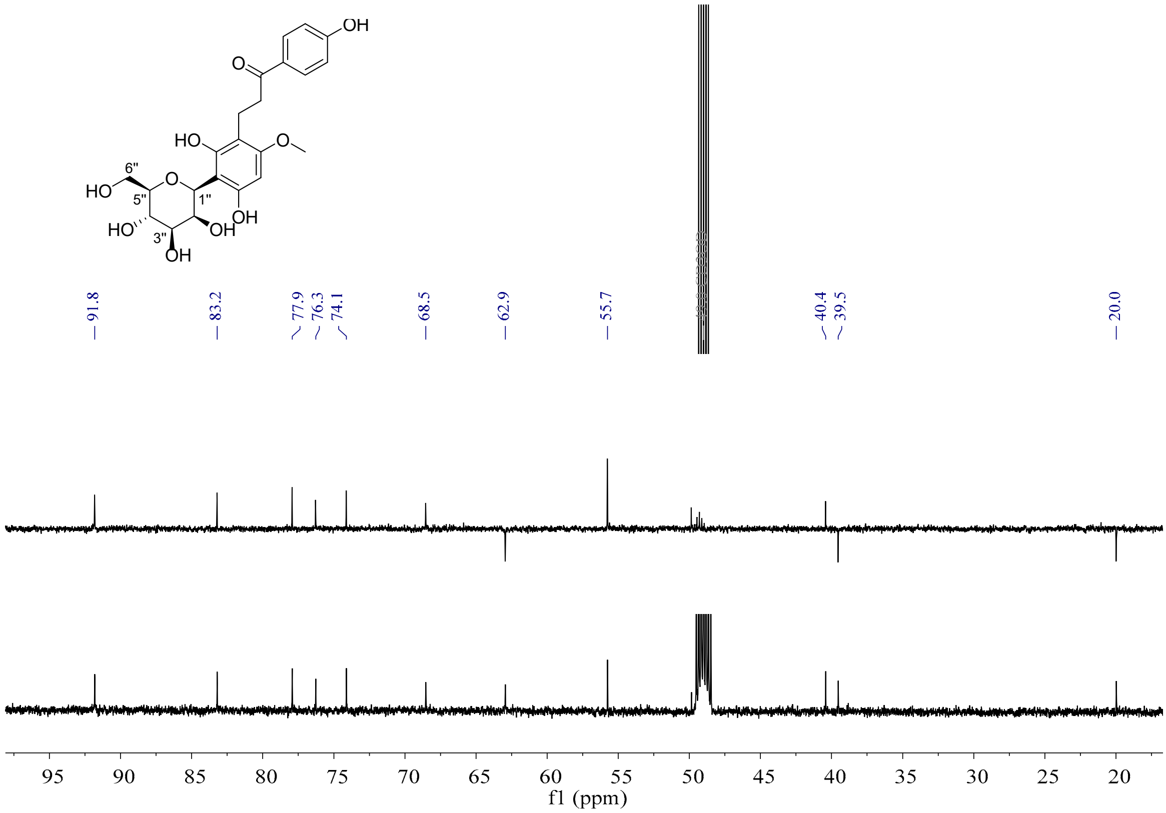

Figure S37. ^{13}C NMR and DEPT spectrum of xuejiein I (**5**) in CD3OD.

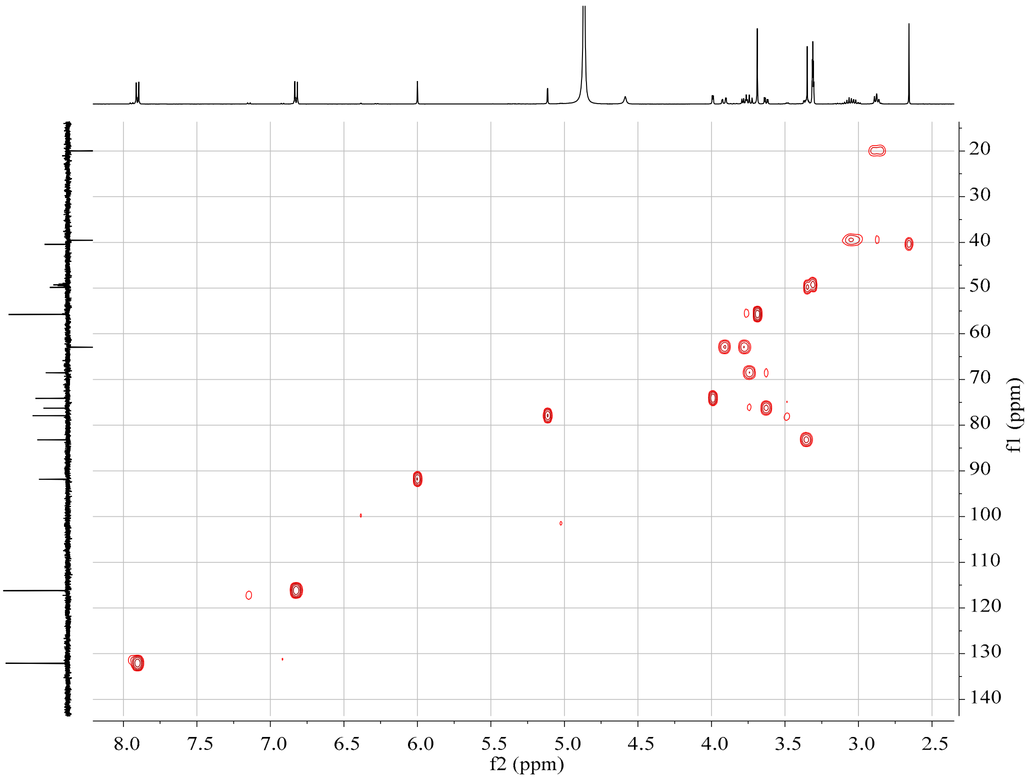

Figure S38. HSQC spectrum of xuejiein I (**5**) in CD_3OD.

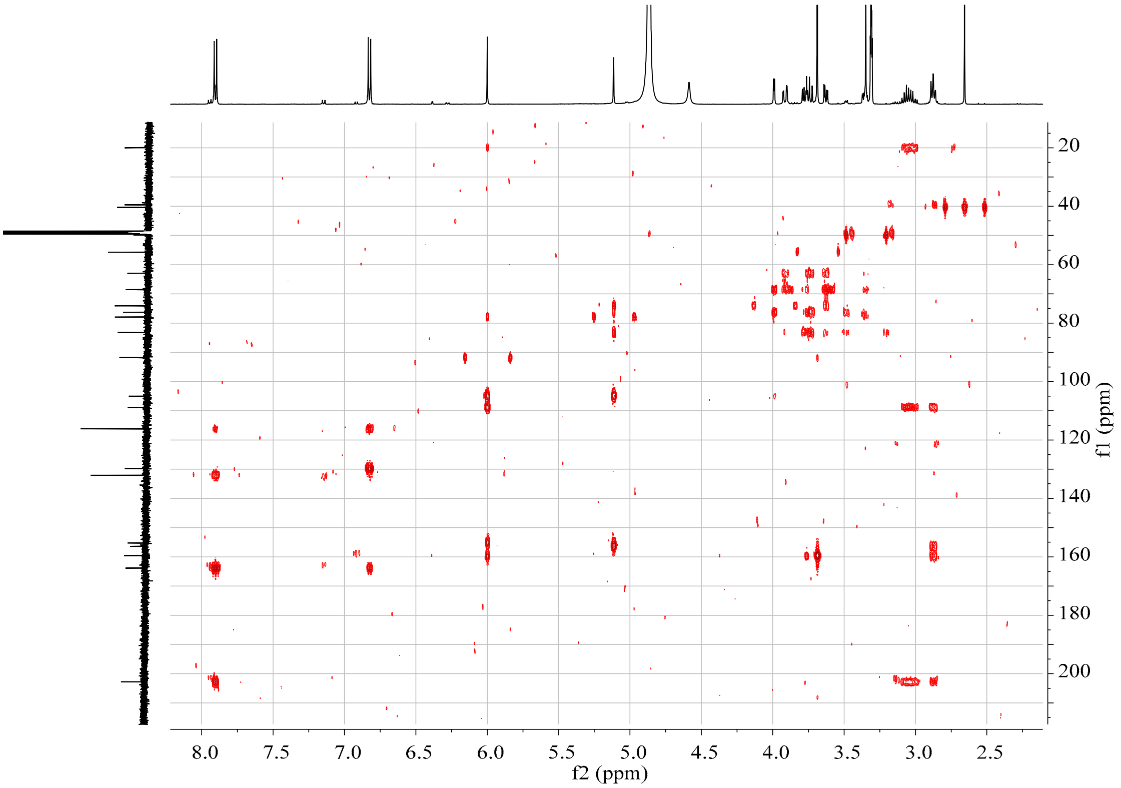

Figure S39. HMBC spectrum of xuejiein I (**5**) in CD_3OD.

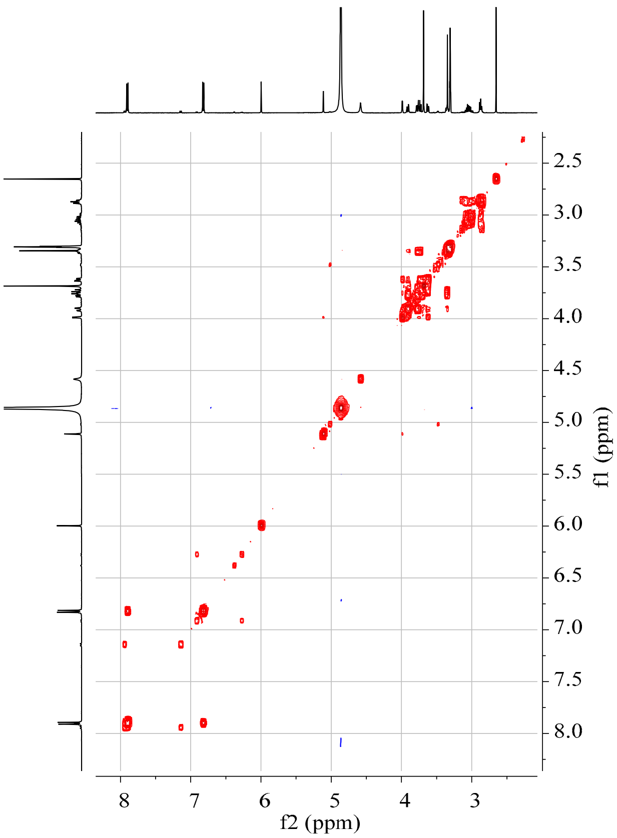

Figure S40. ^{1}H–^{1}H COSY spectrum of xuejiein I (**5**) in CD_3OD.

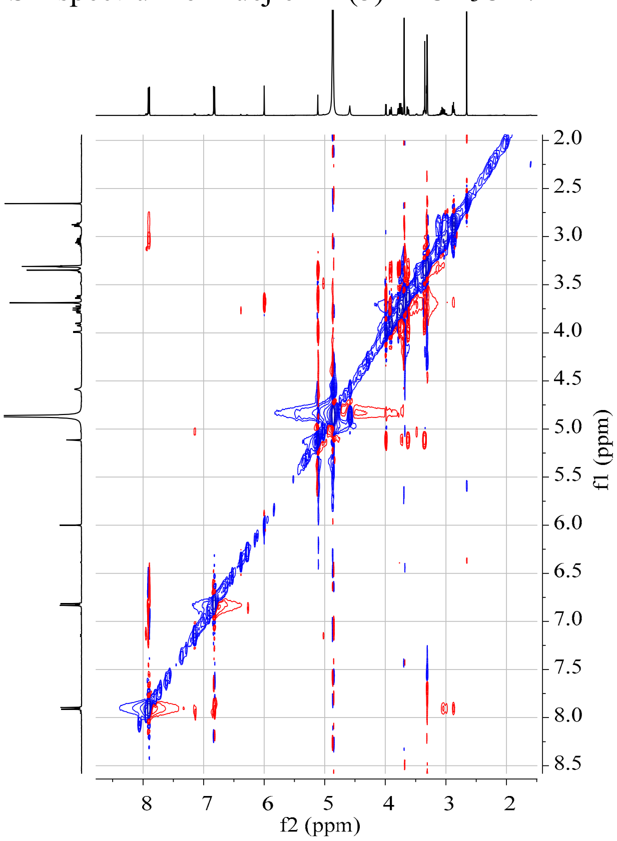

Figure S41. ROESY spectrum of xuejiein I (**5**) in CD_3OD.

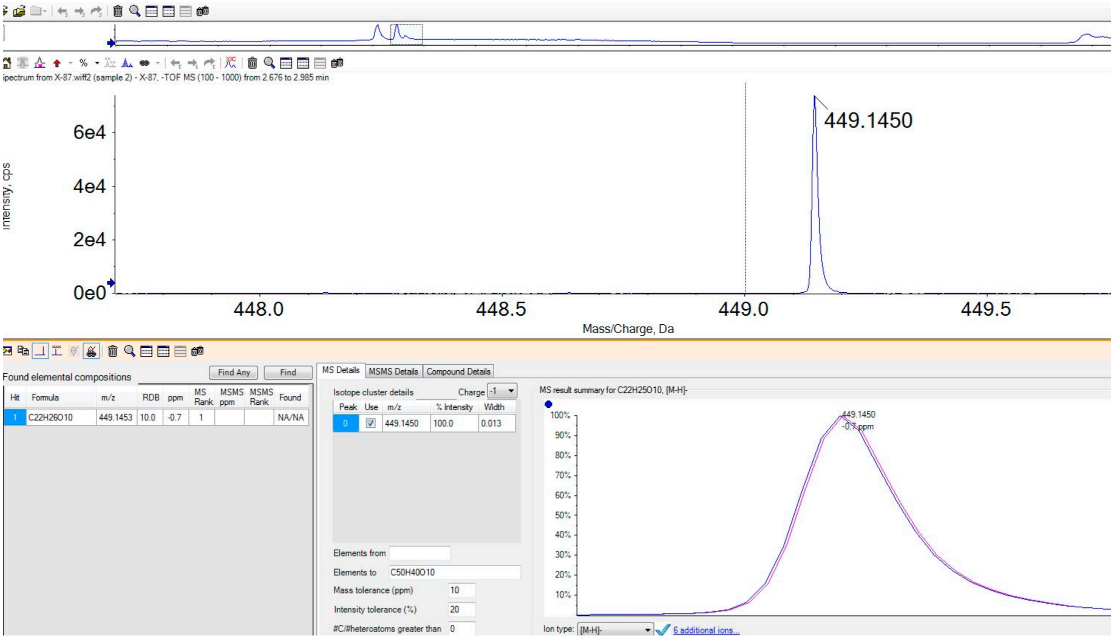

Figure S42. (−)-HRESIMS of xuejiein I (**5**).

4 Computational Calculations

4.1 Conformational Search Geometry Optimization and Frequency Calculations

Conformational searches were conducted with the torsional sampling (Monte Carlo Multiple Minimum, MCMM) method under an OPLS3 [25] force field by the Macromodel 10.2 program (Schrödinger Release 2015-2: MacroModel, Schrödinger, LLC, New York, NY, USA). The value of the 'Energy window for saving structures' was set as 12.6 kcal/mol. All the candidate conformers were subjected to geometry optimization at the B3LYP/6-31G* level of theory in the corresponding solvents applied in the ECD experiments with IEFPCM solvent model, followed by frequency calculations to compute the Gibbs free energies and ensure that all geometries to be at local minima. All quantum chemical calculations were executed in Gaussian 16 program package [26].

4.2 NMR and DP4+ Calculations

NMR shielding tensors were computed at the PCM/mPW1PW91/6-311G** level in chloroform, with the GIAO (gauge-independent atomic orbital) method. To calculate NMR shifts for a particular species, the shielding tensors were first averaged over symmetry-related positions in each conformer and then subjected to Boltzmann averaging over conformers *i* according to eqn (1):

$$\sigma^x = \frac{\sum_i \sigma_i^x \exp(-E_i/RT)}{\sum_i \exp(-E_i/RT)} \qquad (1)$$

where σ^x is the Boltzmann-averaged shielding tensor for nucleus *x* (over all significantly populated conformations), σ_i^x is the shielding tensor for nucleus *x* in conformer *i*, and E_i is the relative Gibbs free energy of conformer *i* (relative to global minimum), obtained from frequency calculations. *R* is the molar gas constant (8.3145 J $K^{-1}mol^{-1}$). The temperature *T* was taken as 298 K.

After Boltzmann weighting conversion of the NMR shielding tensors, the unscaled chemical shifts were computed using TMS as reference standard according to eqn (2):

$$\delta_u = \sigma_0 - \sigma^x \qquad (2)$$

where δ_u is the unscaled chemical shift for nucleus x, σ^x is the Boltzmann-averaged shielding tensor for nucleus x, and σ_0 is the shielding tensor of TMS computed at the same level of theory employed for σ^x.

The unscaled chemical shifts were subjected to DP4+ calculations.

4.3 ECD Calculations

The TDDFT calculations were performed at the PCM/ωB97XD/6-311G** level of theory in methanol. The Boltzmann-averaged ECD spectra were obtained with SpecDis 1.71 [27–29]. The σ and UV correction values applied for the final calculated ECD spectra for compounds **1–5**, which are within a reasonable range.

References:

[25] Harder, E.; Damm, W.; Maple, J.; Wu, C.; Reboul, M.; Xiang, J. Y.; Wang, L.; Lupyan, D.; Dahlgren, M. K.; Knight, J. L.; Kaus, J. W.; Cerutti, D. S.; Krilov, G.; Jorgensen, W. L.; Abel, R.; Friesner, R. A. *Journal of Chemical Theory & Computation* **2016**, *12*, 281–296.

[26] Frisch, M. J.; Trucks, G. W.; Schlegel, H. B.; Scuseria, G. E.; Robb, M. A.; Cheeseman, J. R.; Scalmani, G.; Barone, V.; Petersson, G. A.; Nakatsuji, H.; Li, X.; Caricato, M.; Marenich, A. V.; Bloino, J.; Janesko, B. G.; Gomperts, R.; Mennucci, B.; Hratchian, H. P.; Ortiz, J. V.; Izmaylov, A. F.; Sonnenberg, J. L.; Williams; Ding, F.; Lipparini, F.; Egidi, F.; Goings, J.; Peng, B.; Petrone, A.; Henderson, T.; Ranasinghe, D.; Zakrzewski, V. G.; Gao, J.; Rega, N.; Zheng, G.; Liang, W.; Hada, M.; Ehara, M.; oyota, K.; Fukuda, R.; Hasegawa, J.; Ishida, M.; Nakajima, T.; Honda, Y.; Kitao, O.; Nakai, H.; Vreven, T.; Throssell, K.; Jr, J. A. M.; Peralta, J. E.; Ogliaro, F.; Bearpark, M. J.; Heyd, J. J.; Brothers, E. N.; Kudin, K. N.; Staroverov, V. N.; Keith, T. A.; Kobayashi, R.; Normand, J.; Raghavachari, K.; Rendell, A. P.; Burant, J. C.; Iyengar, S. S.; Tomasi, J.; Cossi, M.; Millam, J. M.; Klene, M.; Adamo, C.; Cammi, R.; Ochterski, J. W.; Martin, R. L.; Morokuma, K.; Farkas, O.; Foresman, J. B.; Fox, D. J. **2016**.

[27] Bruhn, T.; Schaumlöffel, A.; Hemberger, Y.; Bringmann, G. *Chirality* **2013**, *25*, 243–249.

[28] Bruhn, T.; Schaumlöffel, A.; Hemberger, Y.; Pescitelli, G. **2017**.

[29] Pescitelli, G.; Bruhn, T. *Chirality* **2016**, *28*, 466–474.

Figure S43 Experimental and calculated chemical shifts of **1** and its possible isomers

	A	B	C	D	E	F	G	H
1	Functional		Solvent?		Basis Set		Type of Data	
2	mPW1PW91		PCM		6-31+G(d,p)		Unscaled Shifts	
3								
12			DP4+	100.00%	0.00%	-	-	-
14	Nuclei	sp2?	Experimenta	Isomer 1	Isomer 2	Isomer 3	Isomer 4	Isomer 5
15	C	x	141.2	139.6	142.1			
16	C	x	113.7	112.5	112.5			
17	C	x	156.6	153.1	153.4			
18	C	x	101.3	96.9	96.3			
19	C	x	156.5	152.9	153.3			
20	C	x	107.5	104.1	101.2			
21	C		54.9	59.1	50.9			
22	C		58.4	60.3	54.3			
23	C	x	138	137.7	136.5			
24	C	x	130.4	129.4	129.0			
25	C	x	116.1	112.0	113.5			
26	C	x	156.8	153.30	153.30			
27	C	x	116.1	113.00	112.20			
28	C	x	130.4	130.50	128.60			
29	C	x	136.7	132.30	134.70			
30	C	x	145.5	141.70	142.10			
31	C	x	139.8	138.80	138.70			
32	C	x	149.7	144.90	145.00			
33	C	x	105.3	100.20	97.90			
34	C	x	131.8	134.70	130.70			
35	C		51.9	55.80	54.90			
36	C		70.4	72.10	67.30			
37	C		30.8	33.30	34.60			
38	C		60	57.50	57.50			
39	C		56	56.10	56.00			
40								
41	H	x	6.13	6.29	6.27			
42	H	x	5.88	5.57	6.37			
43	H		3.64	3.82	4.14			
44	H		4.2	4.21	4.34			
45	H	x	7.01	7.49	7.86			
46	H	x	6.76	7.05	7.32			
47	H	x	6.76	7.33	7.04			
48	H	x	7.01	7.58	7.79			
49	H	x	6.84	7.11	6.97			
50	H		3.3	3.13	3.13			
51	H		3.94	3.75	4.97			
52	H		2.86	3.17	3.02			
53	H		2.53	2.45	2.97			
54	H		3.23	3.16	3.09			
55	H		3.89	4.03	3.99			

Figure S44. Detailed DP4+ probabilities of all isomers for compound **1**.

	A	B	C	D	E	F	G	H
1	Functional		Solvent?		Basis Set		Type of Data	
2	mPW1PW91		PCM		6-31+G(d,p)		Unscaled Shifts	
3								
4			Isomer 1	Isomer 2	Isomer 3	Isomer 4	Isomer 5	Isomer 6
5	sDP4+ (H data)		100.00%	0.00%	–	–	–	–
6	sDP4+ (C data)		70.23%	29.77%	–	–	–	–
7	sDP4+ (all data)		100.00%	0.00%	–	–	–	–
8	uDP4+ (H data)		100.00%	0.00%	–	–	–	–
9	uDP4+ (C data)		100.00%	0.00%	–	–	–	–
10	uDP4+ (all data)		100.00%	0.00%	–	–	–	–
11	DP4+ (H data)		100.00%	0.00%	–	–	–	–
12	DP4+ (C data)		100.00%	0.00%	–	–	–	–
13	DP4+ (all data)		100.00%	0.00%	–	–	–	–

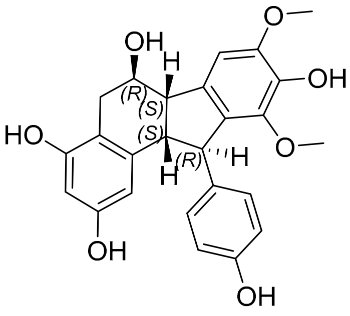

isomer 1-1

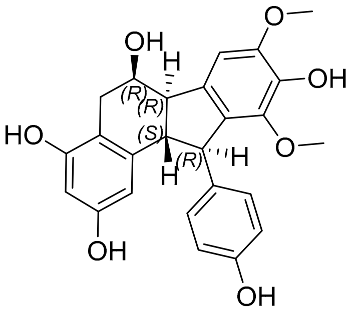

isomer 1-2

5 Bioassays

5.1 Cell Culture and Viability Assay

BV2 mouse microglial cells were cultured and maintained in DMEM/F-12 medium supplemented with 10% fetal bovine serum, 100 U/mL penicillin, and 100 μg/mL streptomycin at 37 °C in a humidified 5% CO_2 incubator. For viability assessment, cells were seeded in 96-well plates at a density of 1 × 10^4 cells/mL in complete medium. After overnight attachment, they were treated with various concentrations of the test compounds for 24 h. Cell viability was then assessed using the Cell Counting Kit-8 (CCK-8) according to the manufacturer's instructions. Briefly, following the addition of CCK-8 solution to each well and incubation at 37 °C for 1 h, the absorbance at 450 nm was measured using a BioTek microplate reader.

5.2 In Vivo Experiments

All procedures involving animals were approved by the Animal Ethics and Welfare Committee of Shenzhen University (Approval No: IACUC-202400148). Eight-week-old male C57BL/6 mice were housed in plastic cages under controlled temperature (24–26°C) and humidity (60%) for one week of acclimatization. The mice were randomly divided into five groups: a normal control (NC) group injected with saline (100 μL/10 g, i.p.); an LPS group receiving LPS (1 mg/kg, i.p.) for two days; an LPS + (−)-**1** group administered compound (−)-**1** (2 mg/kg, nasal administration) 4 hours after the second LPS injection; an LPS + (+)-**1** group administered compound (+)-**1** (2 mg/kg, nasal administration) 4 hours after the second LPS injection; and an LPS + fluoxetine group pretreated daily with fluoxetine (10 mg/kg, i.p.) 2 hours before LPS administration. Depressive-like behaviors were evaluated 1 h post-treatment using the sucrose preference test (SPT), forced swimming test (FST), tail suspension test (TST), and open field test (OFT) in accordance with published methods [30].

[30] Li, W.; Ali, T.; Zheng, C. Y.; He, K. W.; Liu, Z. Z.; Shah, F. A.; Li, N. N.; Yu, Z. J.; Li, S. P. *Molecular Psychiatry* **2022**, *27*, 1047–1058.

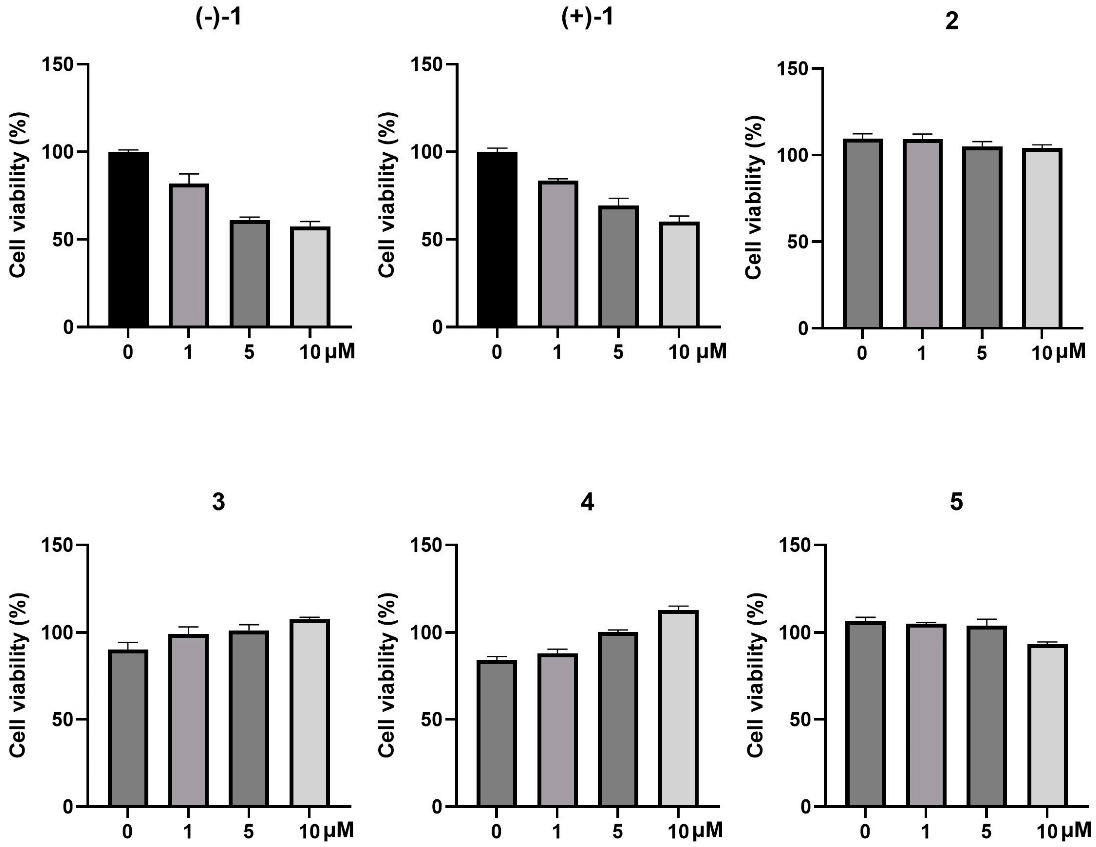

Figure S45 The cell viability of BV2 cells treated with various compound concentrations was measured using the CCK-8 assay, with three replicate wells per concentration.
